# Interaction between polymorphisms in aspirin metabolic pathways, regular aspirin use and colorectal cancer risk: A case-control study in unselected white European populations

**DOI:** 10.1371/journal.pone.0192223

**Published:** 2018-02-09

**Authors:** Harsh Sheth, Emma Northwood, Cornelia M. Ulrich, Dominique Scherer, Faye Elliott, Jennifer H. Barrett, David Forman, C. Roland Wolf, Gillian Smith, Michael S. Jackson, Mauro Santibanez-Koref, Robert Haile, Graham Casey, Mark Jenkins, Aung Ko Win, John L. Hopper, Loic Le Marchand, Noralane M. Lindor, Stephen N. Thibodeau, John D. Potter, John Burn, D. Timothy Bishop

**Affiliations:** 1 Institute of Genetic Medicine, Newcastle University, Newcastle upon Tyne, United Kingdom; 2 Leeds Institute of Cancer and Pathology, University of Leeds, Leeds, United Kingdom; 3 Huntsman Cancer Institute, University of Utah, Salt Lake City, Utah, United States of America; 4 Institute of Medical Biometry and Informatics, University of Heidelberg, Heidelberg, Germany; 5 School of Medicine, University of Dundee, Dundee, United Kingdom; 6 Stanford Cancer Institute, Stanford, California, United States of America; 7 Center for Public Health Genomics, University of Virginia, Charlottesville, Virginia, United States of America; 8 Melbourne School of Population and Global Health, The University of Melbourne, Carlton, Australia; 9 University of Hawaii, Manoa, Hawaii, United States of America; 10 Mayo Clinic, Scottsdale, Arizona, United States of America; 11 Mayo Clinic, Rochester, Minnesota, United States of America; 12 Centre for Public Health Research, Massey University, Wellington, New Zealand; National Institute of Environmental Health Sciences, UNITED STATES

## Abstract

Regular aspirin use is associated with reduced risk of colorectal cancer (CRC). Variation in aspirin’s chemoprevention efficacy has been attributed to the presence of single nucleotide polymorphisms (SNPs). We conducted a meta-analysis using two large population-based case-control datasets, the UK-Leeds Colorectal Cancer Study Group and the NIH-Colon Cancer Family Registry, having a combined total of 3325 cases and 2262 controls. The aim was to assess 42 candidate SNPs in 15 genes whose association with colorectal cancer risk was putatively modified by aspirin use, in the literature. Log odds ratios (ORs) and standard errors were estimated for each dataset separately using logistic regression adjusting for age, sex and study site, and dataset-specific results were combined using random effects meta-analysis. Meta-analysis showed association between SNPs rs6983267, rs11694911 and rs2302615 with CRC risk reduction (All *P*<0.05). Association for SNP rs6983267 in the *CCAT2* gene only was noteworthy after multiple test correction (*P* = 0.001). Site-specific analysis showed association between SNPs rs1799853 and rs2302615 with reduced colon cancer risk only (*P =* 0.01 and *P* = 0.004, respectively), however neither reached significance threshold following multiple test correction. Meta-analysis of SNPs rs2070959 and rs1105879 in *UGT1A6* gene showed interaction between aspirin use and CRC risk (*P*_interaction_ = 0.01 and 0.02, respectively); stratification by aspirin use showed an association for decreased CRC risk for aspirin users having a wild-type genotype (rs2070959 OR = 0.77, 95% CI = 0.68–0.86; rs1105879 OR = 0.77 95% CI = 0.69–0.86) compared to variant allele cariers. The direction of the interaction however is in contrast to that published in studies on colorectal adenomas. Both SNPs showed potential site-specific interaction with aspirin use and colon cancer risk only (*P*_interaction_ = 0.006 and 0.008, respectively), with the direction of association similar to that observed for CRC. Additionally, they showed interaction between any non-steroidal anti-inflammatory drugs (including aspirin) use and CRC risk (*P*_interaction_ = 0.01 for both). All gene x environment (GxE) interactions however were not significant after multiple test correction. Candidate gene investigation indicated no evidence of GxE interaction between genetic variants in genes involved in aspirin pathways, regular aspirin use and colorectal cancer risk.

## Introduction

Observational studies have consistently demonstrated an association between regular aspirin or other non-steroidal anti-inflammatory drug (NSAID) use and reduced colorectal cancer (CRC) risk [1]. For CRC, a randomized chemopreventive trial in patients with Lynch syndrome showed beneficial effect of aspirin use in reducing CRC risk, and in the general population, long-term risk reduction is most evident from follow-up of the cardiovascular disease prevention trials conducted in the 1980s [1–4]. Optimizing chemoprevention requires understanding the factors that limit the full impact of aspirin dosage. Advances in elucidating aspirin’s mode of action on cellular pathways within the colonic epithelium have been made; in particular- inhibition of cyclooxygenase activity, inhibition of NF-κB, induction of polyamine catabolism and downregulation of WNT-β-catenin signalling [5–9]. Although, further work is required to fully clarify these mechanisms. Delineating interaction of aspirin and its metabolites with molecules involved in key cellular processes associated with tumorigenesis could not only help validate variation in the chemopreventive efficacy but also aid in understanding the neoplastic transformation of colonic epithelial cells [10]. Furthermore, identifying gene x environment (GxE) interactions between genetic markers and aspirin use could help in stratifying individuals who would most benefit (or not benefit) from taking aspirin.

Inter-individual variation in the chemopreventive effect on colorectal neoplasia has been in part attributed to germline variations, particularly single nucleotide polymorphisms (SNPs). Whilst a large number of SNPs in aspirin’s pharmacokinetic and pharmacodynamic pathways have been suggested to modulate chemopreventive effect in observational studies [6, 9, 11–17], these associations have not been replicated. Furthermore, a large genome-wide association study (GWAS) carried out Nan *et al* 2015, investigated potential GxE interactions based on regular use of any NSAIDs (including aspirin) as exposure [6]. Whilst, such GWAS studies offer a “hypothesis-free” approach to test for association between variants and phenotype of interest, the statistical cost of extreme multiple testing compromises power; candidate gene approach utilises an *a priori* biologically-motivated hypothesis to assess association between variants in putative genes and phenotype of interest. Hence, the techniques are complementary approaches to identifying potential sources of GxE interactions within observational studies.

To our knowledge, no meta-analysis has been reported in the literature which tested for GxE interaction between SNPs in aspirin pathway genes, regular aspirin use and CRC risk. Therefore, the current study aimed to assess associations of candidate SNPs that putatively modify aspirin’s pharmacological effects by carrying out random effects meta-analysis of GxE interaction between aspirin only use and CRC risk using two large population based case-control datasets: UK-Colorectal Cancer Study Group (UK-CCSG) and NIH- Colon Cancer Family Registry (NIH-CCFR).

## Materials and methods

### Study samples and epidemiological questionnaire

Individual level data from the UK-CCSG (recruitment 1997–2013) and NIH-CCFR Phase I (recruitment in Australia, Canada and the USA, 1997–2002) case-control datasets were pooled. Both studies enrolled incident CRC cases and healthy population controls [18, 19].

From the UK-CCSG dataset, CRC cases and controls from all three study sites (Leeds, Dundee and York) were included in the analysis. Briefly, cases between the age of 45 and 80 years with histologically confirmed incident CRC and diagnosed in the period of 1997–2000, were identified at all three sites while for 2000–2013 only from Leeds. Patients who had a primary cancer previously, history of coeliac disease, familial adenomatous polyposis, diverticular disease 2 years before current cancer diagnosis, non-adenocarcinoma colorectal cancer or ulcerative colitis diagnosed in previous 3 years were not recruited in the study. Healthy population based controls were identified through patient’s GP practice list. An age and sex matched control with no history of previous cancer at the time of recruitment was identified for each case between 1997 and 2000 at all 3 sites, and following 2000, friends or spouse of cases from Leeds site with no history of cancer at the time of recruitment were collected for the study to complement GP recruitment.

From the NIH-CCFR dataset, CRC cases and controls from three study sites (Ontario, Melbourne and Fred Hutchinson Cancer Research Centre) were included in the analysis. Despite availability of epidemiological data on cases and controls from the three other NIH-CCFR sites (University of Southern California, Mayo Clinic and Hawaii), these sites were excluded from the analysis due to the absence of genome-wide SNP genotype data for controls. For the current study, incident case proband identified through population based cancer registries recruited between 1997 and 2002 were included in the analysis. Healthy population based and spouse controls were identified through medicare and driver’s license files, telephone subscriber lists and electoral rolls and were randomly selected between 1997 and 2002.

For the UK-CCSG dataset, a research nurse carried out interviews of the study participants either in hospital or at home. All participants completed detailed diet and lifestyle questionnaire called the Food Frequency and Epidemiology Questionnaire [19], which was modelled on the questionnaire developed and validated by the European Prospective Investigation into Cancer and Nutrition [20]. Anonymised UK-CCSG raw dataset is present in S1 File. For the NIH-CCFR dataset, each study participant completed a standardised family history, personal exposure and baseline epidemiologic questionnaire either in person (University of Southern California site), by telephone (Fred Hutchinson cancer research Centre, University of Southern California and University of Queensland Melbourne sites) or by mail (University of Hawaii, Cancer Care Ontario and Mayo Clinic sites) [18]. Questionnaires were customised by the participating centres for local usage, in particular for different language conventions and brand names, and added some questions of local interests. Copy of the phase 1 baseline family history and baseline epidemiologic questionnaires from all 6 sites can be downloaded from: http://coloncfr.org/questionnaires.

All participants provided written informed consent at the time of the interview and the study design was approved by the Institutional Review Boards at each NIH-CCFR site. The UK-CCSG study as well as the joint analysis of the UK-CCSG and NIH-CCFR data were approved by the Leeds East Research Ethics Committee.

Because of sample numbers, only self-reported non-Hispanic white individuals were included in the analysis. A baseline epidemiologic questionnaire containing details about medical history and medication use (including use of aspirin, NSAIDs or both) was completed by participants in both studies; the measurement instrument differed between the studies overall but were equivalent for assessing NSAID usage (S1 Table).

### SNP selection and genotyping

Literature review was carried out using PubMed and Google Scholar to identify SNPs in putative genes. A combination of keywords such as- “aspirin”, “NSAID”, “pharmacogenetics”, “polymorphisms”, “SNPs”, “gene variant”, “colorectal adenoma” and “colorectal cancer”, was used to search for relevant literature published between 1990 and 2015 in the search engines. On PubMed, this resulted in 103 abstracts. All studies presenting original data on SNP and colorectal adenoma or carcinoma risk association, or interaction between SNP and aspirin (or NSAID) use in relation to colorectal adenoma or carcinoma were retrieved and reviewed. Types of articles reviewed included original article, editorial letter, conference abstract, observational study, meta-analysis, review, systemic review and randomized controlled trial. The UK-CCSG study collaborators crosschecked candidate SNP selection from the literature. From the published studies reviewed until May 2015, a total of 42 SNPs from 15 candidate genes were selected for analysis (S2 Table).

We selected genes and SNPs as follows: (i) from previous studies of colorectal adenoma or cancer where statistical evidence–after correction for multiple testing–of an interaction with NSAID use (including aspirin) had been observed (*ALOX15*, *IL16*, *MDR1*, *MGST1*, *NFkB*, *UGT1A6*, *PTGS1* and *PTGS2*) [6, 9, 12, 14, 15, 21–23]; (ii) SNPs in the genes *CES2* and *PAFAH1B2* were selected as these genes have been shown to metabolize aspirin in intestine and blood, respectively, but had not been tested for interaction with aspirin use in relation to CRC risk previously [24, 25]; (iii) SNPs in genes involved in the metabolism of aspirin that have been shown to be associated with colorectal cancer or adenoma risk (*CYP2C9*) [26] and; (iv) SNPs in genes that are direct or indirect targets of aspirin and have been shown to be associated with colorectal cancer or adenoma risk (*IkBkB*, *CCAT2*, 20p12 locus, *NCF4* and *ODC1*) [6, 8, 13, 16, 27].

No SNPs were genotyped for the NIH-CCFR dataset as these samples had been the subject of genome-wide genotyping [28] using Illumina Human 1M, IM Duo and Omni1 arrays (S3 Table). Taqman allelic discrimination assay (Applied Biosystems, Paisley, UK) was used for the UK-CCSG sample set to supplement genotyping with an Illumina HumanExome BeadChip array v1.1 (Illumina, San Diego, USA) (S3 Table). A detailed description of genotyping, quality assurance and control, and imputation is provided in S2 File.

SNPs were excluded if they were tri-allelic; had a call rate of <98%; had a minor allele frequency (MAF) of <4%; or there was evidence of being out of Hardy Weinberg equilibrium (HWE) in controls (*P*<0.001 after multiple tests correction). Overall, 15 of the 42 SNPs were removed from analysis as the observed MAF was <4%. For the remaining SNPs, all were in HWE and the MAF between the two datasets was similar (S4 Table).

### Statistical analysis

Common data elements were defined for both datasets to produce common definitions and coding terms (S1 Table). Continuous variables such as BMI, alcohol and physical activity were converted to dichotomous variables by a median split. Comparison of SNP minor allele frequency between controls in two datasets was carried out using Fisher’s exact test (S4 Table). SNPs within the same chromosome were tested for linkage disequilibrium (R^2^) in controls in both datasets (S1 and S2 Figs). Each genotyped SNP was coded as 0, 1, or 2 for the number of copies of minor allele and imputed SNP, such as rs20417 in the NIH-CCFR dataset, was coded based on the “expected” number of copies of the minor allele.

The odds ratio (OR) and 95% confidence interval (CI) for associations between known epidemiological risk factors, “aspirin only” use, and SNP genotype with CRC risk were assessed using logistic regression models adjusted for age, sex and study site separately for each study. Each SNP was tested for association with colorectal cancer risk using logistic regression and assuming an additive model of inheritance. The interaction of SNP genotype with “aspirin only” use in relation to CRC risk was investigated. Participants who took aspirin only were included in the analysis, whereas, users of both aspirin and other NSAIDs (UK-CCSG n = 49, NIH-CCFR n = 160) or other NSAIDs only (UK-CCSG n = 258, NIH-CCFR n = 261) were excluded from the analysis. Regular aspirin use was defined as daily aspirin intake for 3 months or longer in the UK-CCSG dataset whereas it was defined as twice a week aspirin intake for more than a month in the NIH-CCFR dataset (S1 Table). GxE interaction was tested using logistic regression of the cross product of the presence of variant allele and dichotomous regular use of aspirin-only. The likelihood ratio test was used to assess the statistical significance of the interaction with adjustment for age, sex and study site.

Meta-analysis of the association and interaction tests for the SNPs and “aspirin only” use with CRC risk in both datasets was carried out. Estimates of log odds ratios and standard error from both datasets, which were adjusted for age, sex and study site, were used to calculate combined risk estimates using random-effects models where the log OR were weighted by the method of DerSimonian and Laird [29]. To test for heterogeneity between the estimates from the two datasets, Cochran’s Q-test and Higgin’s I-squared statistic [30] was calculated. All tests were carried out using an additive inheritance model. All *P*-values were two-sided and the significance threshold was set at ≤0.001 to allow for multiple testing (Bonferroni correction for 42 SNPs, 0.05/ 42 = 0.001). All analyses were conducted in Stata V12 (Stata Corp., College Station, USA). Checklist outlining information about the justification for the study and the methodology employed is provided in S3 File.

## Results

### Baseline epidemiological characteristics of cases and controls

Overall, the UK-CCSG dataset consisted of 1910 cases and 1275 controls and the NIH-CCFR dataset consisted of 1415 cases and 986 controls. Characteristics of the study population are presented in Table 1 (Further information in S5 Table). Compared to the UK-CCSG dataset, the NIH-CCFR dataset had higher proportion of cases under the age of 50 (Table 1), resulting from the differences in case ascertainment strategies. Association of known epidemiological risk factors, including aspirin use, with CRC risk in both studies was consistent with the existing literature (Table 1). Due to the availability of participant’s data on weight at the age 20 in both datasets, we tested for association between BMI at age 20 and CRC risk. We observed a positive association between BMI and CRC risk which is consistent with a prospective study, which had shown that weight gain during early adulthood increased risk of colon cancer [31]. Association between family history and CRC risk in the NIH-CCFR dataset was not tested since the study sites used different case recruitment strategies, some of which selected cases based on the family history and age at cancer diagnosis [18].

**Table 1:** Characteristics of population based UK-Colorectal Cancer Study Group (UK-CCSG) and NIH-Colon Cancer Family Registry (NIH-CCFR) datasets. For odds ratio comparisons, the baseline level is indicated.

	UK-Colorectal Cancer Study Group (N = 3185)				NIH-Colon Cancer Family Registry (N = 2401)			
	Controls (n = 1275), n (%)	Cases (n = 1910), n (%)	Odds Ratio (95% CI)	*P-*value	Controls (n = 986), n (%)	Cases (n = 1415), n (%)	Odds Ratio (95% CI)	*P-*value
**Sex, n(%)**								
Male (baseline)	635 (49.8)	1124 (58.8)			473 (48.0)	731 (51.7)		
Female	639 (50.2)	786 (41.2)	0.69 (0.60–0.80)	<0.001	513 (52.0)	684 (48.3)	0.86 (0.73–1.02)	0.08
**Age (in years)**^**a**^**, n (%)**								
<50	42 (3.3)	106 (5.6)	-	-	154 (15.6)	637 (45.0)	-	-
50–59	167 (13.1)	309 (16.2)			299 (30.3)	334 (23.6)		
60–69	493 (38.6)	646 (33.8)			305 (30.9)	314 (22.2)		
70–79	505 (39.6)	685 (35.9)			228 (23.1)	130 (9.2)		
80–89	69 (5.4)	161 (8.4)			-	-		
≥90	-	3 (0.2)			-	-		
**Primary cancer site, n(%)**										
Colon	-	1222 (64.0)	-	-	-	804 (62.6)	-	-
Rectum	-	688 (36.0)			-	480 (37.4)		
**BMI at 20 years**^**b**^**, n(%)**								
Low (baseline)	628 (49.9)	821 (44.8)			481 (49.5)	575 (41.3)		
High	630 (50.1)	1013 (55.2)	1.23 (1.07–1.42)	0.005	490 (50.5)	816 (58.7)	1.39 (1.18–1.64)	<0.0001
**Family history of cancer**^**c**^**, n(%)**								
No (baseline)	209 (47.4)	195 (29.5)			-	-		
First or (and) second degree relative	232 (52.6)	466 (70.5)	2.15 (1.68–2.77)	1.98 x 10^−9^	-	-	-	^-^
**Cigarette smoking**^**d**^**, n(%)**								
No (baseline)	555 (43.7)	710 (37.6)			408 (41.4)	557 (39.4)		
Yes	715 (56.3)	1179 (62.4)	1.29 (1.12–1.49)	0.001	578 (58.6)	856 (60.6)	1.08 (0.92–1.28)	0.34
**Alcohol intake**^**e**^**, n(%)**								
Low (baseline)	642 (50.6)	741 (39.6)			146 (48.2)	219 (42.3)		
High	626 (49.4)	1130 (60.4)	1.56 (1.34–1.81)	1.01 x 10^−9^	157 (51.8)	299 (57.7)	1.27 (0.95–1.69)	0.10
**Physical activity**^**f**^**, n(%)**								
Low (baseline)	622 (49.1)	976 (52.3)			-	-		
High	646 (50.9)	888 (47.7)	0.88 (0.76–1.01)	0.07	-	-	-	-
**Regular aspirin only use**^**g**^**, n(%)**								
No (baseline)	848 (76.8)	1421 (81.4)			518 (64.7)	874 (75.2)		
Yes	256 (23.2)	324 (18.6)	0.76 (0.63–0.91)	0.003	283 (35.3)	531 (24.8)	0.60 (0.50–0.73)	4.80 x 10^−7^
**Regular NSAID use**^**g**^**, n(%)**								
No (baseline)	848 (66.9)	1421 (75.3)			518 (52.9)	874 (62.2)		
Yes	420 (33.1)	467 (24.7)	0.66 (0.57–0.78)	3.58 x 10^−7^	461 (47.1)	531 (37.8)	0.68 (0.58–0.81)	6.04 x 10^−6^

Continuous variables such as BMI, alcohol intake and physical activity were dichotomised by placing a cutoff point at the median split in controls. Odds ratio was calculated using logistic regression and depicts association with colorectal cancer risk.

^a^ Age at the time of diagnosis for cases and interview for controls

^b^ Body Mass Index (BMI) cut-off point in UK-CCSG and NIH-CCFR dataset is 21.8 kg/m^2^ and 21.5 kg/m^2^ respectively. Participants below the cut-off point are categorised as “Low” and above the cut-off point are categorised as “High” in the respective datasets.

^c^ Association between family history of cancer and CRC risk not calculated for the NIH-CCFR dataset due to the different case recruitment strategies employed by the study sites

^d^ “Yes” is defined as ever smoked 1 cigarette a day for 3 months or longer. “No” is defined as never smoked a cigarette.

^e^ Alcohol intake cut off point in UK-CCSG and NIH-CCFR dataset is 5.6 units/day and 0.89 units/day respectively. Participants below the cut-off point are categorised as “Low” and above the cut-off point are categorised as “High” in the respective datasets.

^**f**^ Physical activity cut off point in UK-CCSG dataset 3 hours/week. Participants below the cut-off point are categorised as “Low” and above the cut-off point are categorised as “High” in the respective datasets.

^**g**^ Regular aspirin or NSAID use is defined as regular intake for a period of 3 months or longer in the UK-CCSG dataset whereas it is defined as regular use of at least two pills per week for at least one month in the NIH-CCFR dataset.

### SNP association with CRC risk

Each SNP was assessed for its association with CRC risk using logistic regression separately by dataset (S6 Table). Meta-analysis of the datasets showed a reduced risk of CRC associated with the presence of variant allele of SNPs rs6983267 (OR = 0.86, 95% CI = 0.79–0.94, *P* = 0.001, I^2^ = 0), rs2302615 (OR = 0.85, 95% CI = 0.74–0.98, *P* = 0.02, I^2 =^ 0) and rs11694911 (OR = 0.85, 95% CI = 0.74–0.96, *P* = 0.01, I^2^ = 0) at *CCAT2* and the *ODC1* gene locus, respectively (S7 Table).

Site-specific meta-analysis of colon and rectum showed approximately 20% decrease in colon cancer risk only in the presence of *CYP2C9* rs1799853 variant allele (OR = 0.79, 95% CI = 0.65–0.95, *P* = 0.01, I^2^ = 0) and *ODC1* rs2302615 variant allele (OR = 0.80, 95% CI = 0.69–0.93, *P* = 0.004, I^2^ = 0) (S8 and S9 Tables). No site-specific association was observed for the *CCAT2* rs6983267 variant allele, as it was associated with reduced risk of both colon cancer (OR = 0.86, 95% CI = 0.78–0.95, *P* = 0.002, I^2^ = 0; S9 Table) and rectal cancer (OR = 0.85, 95% CI = 0.75–0.95, *P* = 0.005, I^2^ = 0; S10 Table).

### Gene x aspirin-only use interactions

**Fig 1:**
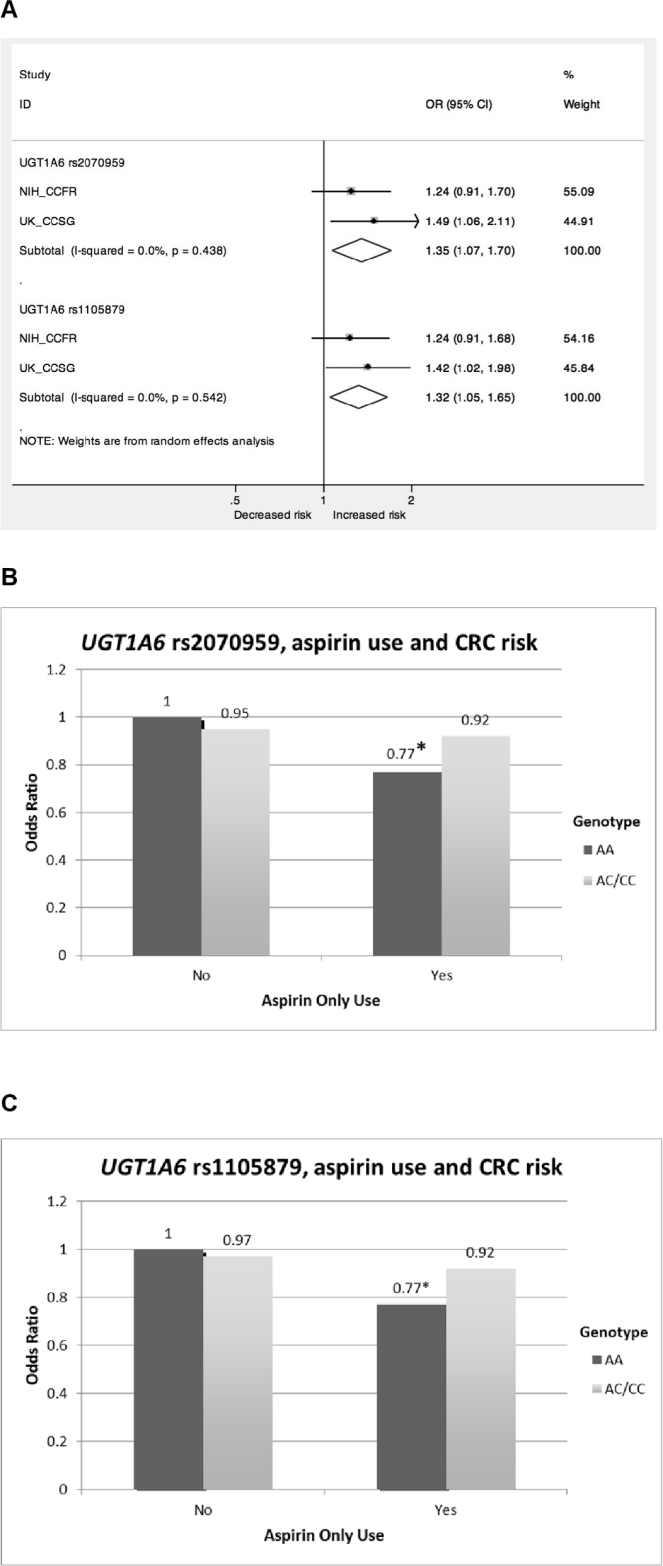
Meta-analysis of interaction between SNP variant allele, aspirin-only use and colorectal cancer risk. (**A**) Forest plot depicting meta-analysis odds ratio of GxE interaction term. I-squared is the measure of the variation in odds ratio attributable to heterogeneity [30] and *P*-value tests for heterogeneity between the UK-CCSG and NIH-CCFR datasets. (**B**) Association between *UGT1A6* SNP rs2070959 (T181A) variant allele and CRC risk stratified by aspirin use. *P*_**interaction**_ = 0.01. (**C**) Association between *UGT1A6* SNP rs1105879 (R184S) variant allele and CRC risk stratified by aspirin use. *P*_**interaction**_ = 0.02. *P-value for association <0.001; UK-CCSG: UK-Colorectal Cancer Study Group; NIH-CCFR: NIH-Colon Cancer Family Registry.

Each SNP was assessed for an interaction with aspirin-only use in relation to CRC risk, separately by dataset and combined in a meta-analysis. In the meta-analysis, SNP rs2070959 in the *UGT1A6* gene showed evidence of an interaction between aspirin-only use and CRC risk (*P*_interaction_ = 0.01, I^2^ = 0), whereby aspirin users with wild-type genotype were associated with 23% lower CRC risk (OR = 0.77, 95% CI = 0.69–0.86) compared to negligible risk reduction in variant allele carriers (OR = 0.92, 95% CI = 0.86–0.99) (Fig 1; S11 and S12 Tables). A second SNP in *UGT1A6* gene: rs1105879, which is in high linkage disequilibrium (LD) with rs2070959 (R^2^ = 0.90), showed similar evidence (Fig 1; S11 and S12 Tables; S1 and S2 Figs). No significant difference in risk reduction was observed between wild-type genotype and variant allele carriers for both SNPs in non-users of aspirin.

**Fig 2:**
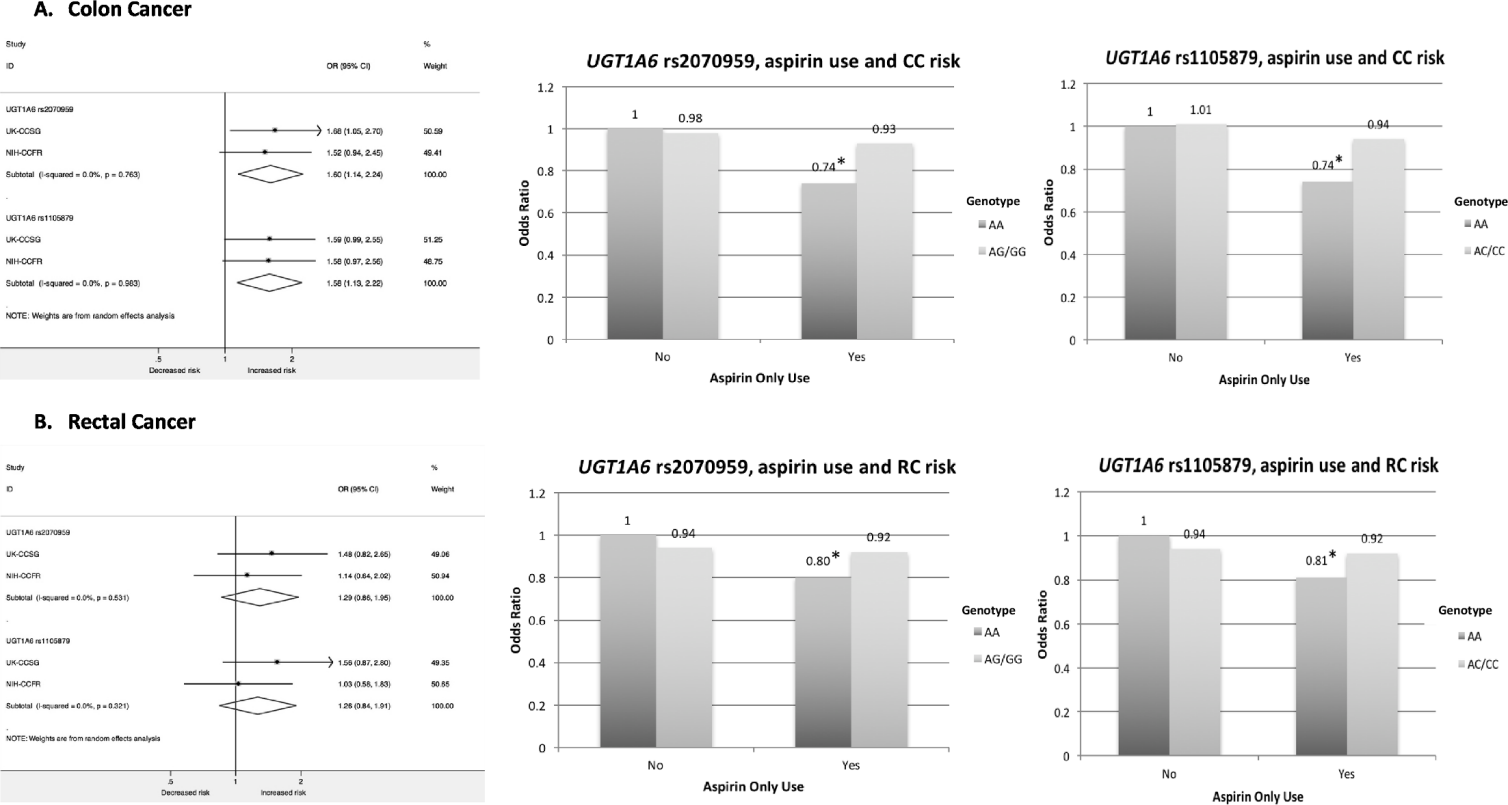
**Meta-analysis of site-specific interaction between SNP variant allele, aspirin-only use and (A) colon cancer risk and (B) rectal cancer risk**. (**A**) Association between *UGT1A6* rs2070959 (T181A) variant allele and colon cancer risk stratified by aspirin use (*P*_interaction_ = 0.006); association between *UGT1A7* rs1105879 (R184S) variant allele and colon cancer risk stratified by aspirin use (*P*_interaction_ = 0.008). (**B**) Association between *UGT1A6* rs2070959 (T181A) variant allele and rectal cancer risk stratified by aspirin use (*P*_interaction_ = 0.22); association between *UGT1A6* rs1105879 (R184S) variant allele and rectal cancer risk stratified by aspirin use (*P*_interaction_ = 0.26). Forest plot depicting meta-analysis odds ratio of gene x environment interaction term. I-squared is the measure of the variation in odds ratio attributable to heterogeneity [30] and *P*-value tests for heterogeneity between the UK-CCSG and NIH-CCFR datasets. *P-value for association <0.05; CC: Colon Cancer; RC: Rectal Cancer; UK-CCSG: UK-Colorectal Cancer Study Group; NIH-CCFR: NIH-Colon Cancer Family Registry.

Meta-analysis of the GxE interaction stratified by tumour site showed an interaction between SNP rs2070959 and aspirin-only use in relation to colon cancer risk (*P*_interaction_ = 0.006): aspirin users with the wild-type genotype showed a 26% lower risk of colon cancer than non-users with wild-type genotype (OR = 0.74, 95% CI = 0.65–0.84), whereas, no difference in risk reduction was observed in variant allele carriers regardless of the aspirin use status (Fig 2A). We observed a similar direction and magnitude of interaction between SNP rs1105879 and aspirin-only use in relation to colon cancer risk (*P*_interaction_ = 0.008). No evidence of interaction was observed between either SNP, aspirin use and rectal cancer risk (Fig 2B). Haplotype analysis of SNPs was not conducted as the SNPs were in high LD and would not have provided additional statistical power to observe any potential interaction (S1 and S2 Figs).

### Sensitivity analysis

The predefined analysis plan included participants of all ages. However, the UK-CCSG cohort represents population-based cases (6.9% of cases below the age of 50 years) whereas the NIH-CCFR cohort is enhanced for early onset (47.7% of cases below the age of 50 years). Since aspirin is prescribed prophylactically for cardiovascular diseases after the age of 50, it is likely that the participants under the age of 50 may not be exposed to aspirin. Hence, we performed sensitivity analysis by excluding all participants under the age of 50 and carried out GxE interaction test between only aspirin use, SNP variant allele and CRC risk. Compared to the analysis carried out in all participants, we observed similar direction and magnitude of interaction between SNPs in the *UGT1A6* genes, only aspirin use and CRC risk age restricted analysis (*P*_interaction_ = 0.009, I^2^ = 0 for rs2070959; *P*_interaction_ = 0.01, I^2^ = 0 for rs1105879; S13 Table).

## Discussion

In the current meta-analysis, we observed association between the SNP at *CCAT2* gene locus with reduced CRC risk, which was noteworthy after multiple test correction and congruent with the literature [26, 32]. Potential site-specific association between rs1799853 in *CYP2C9* and colon cancer risk was complemented by the assessment of the difference in CYP2C9 protein expression between colon and rectum in the Human Protein Atlas. Medium protein expression is observed in the colon but not rectal tissue [33], suggesting that there may be relevant differences in site-specific metabolism. SNP rs1799853 is a nonsynonymous coding variant (p.R144C) which codes for a CYP2C9 enzyme with reduced activity and metabolic capacity [34]. As CYP2C9 enzyme metabolises pro-carcinogenic xenobiotic compounds such as- polycyclic aromatic hydrocarbons and heterocyclic aromatic amines- in the intestinal epithelium, it leads to an increased risk of cancer [35]. Hence, the carriers of the SNP rs1799853 variant allele may observe reduced risk of developing colon cancer compared to rectal cancer.

Interestingly, we observed a potential site-specific association for decrease in colon cancer risk in the presence of *ODC1*’s rs2302615 A allele. Whilst the association was not significant after multiple test correction, it was complemented by the study carried out by Zell *et al*. which observed decreased probability of survival in patients diagnosed with rectal cancer and carrying rs2032615 A allele (ODC1 GA/ AA genotype HR = 2.92, 95% CI = 1.22–7.03) but not in colon cancer patients (ODC1 GA/ AA genotype HR = 1.76, 95% CI = 0.85–3.63) [36]. Ornithine decarboxylase (ODC) regulates polyamine synthesis. Upregulated polyamine metabolism is associated with increased risk of colorectal cancer. Regulation of *ODC1* is carried out by E-box transcription factors, chiefly- transcriptional activator c-MYC and transcriptional repression MAD1. Tissue specific RNA-seq data in the Human Protein Atlas show 7.5 and 5.8 transcripts per million of MAD1 in colon and rectum tissues, suggesting slightly higher expression of MAD1 in the colon compared to rectum [33]. Coupled this with the observation suggesting preferential binding of MAD1 to *ODC1* promoter containing A allele in two colon cancer cell lines- HT29 and HCT116 [36], it is likely that the carriers of rs2302615’s variant allele may observe reduced risk of developing colon cancer.

Upon assessing GxE interaction, aspirin-only users with wild-type genotype of the SNPs in *UGT1A6* gene gave suggestive evidence of decreased risk of CRC or colon cancer but not in variant allele carriers. The UDP glucuronosyltransferase 1A6 (UGT1A6) protein is involved in metabolism of salicylic acid through glucuronidation [37]. A study in liver microsomes and urine analysis in young volunteers following aspirin intervention has shown that homozygous mutant carriers of the SNPs rs2070959 and rs1105879 have a higher metabolic activity than wild-type carriers [37, 38], hence wild-type carriers are likely to benefit from aspirin intervention, consistent with our interaction data. In previous studies, a significant interaction between *UGT1A6* SNPs, aspirin use and colorectal adenoma risk has been observed whereby, carriers of variant allele were associated with lower risk of colorectal adenoma in aspirin users compared to non-users [17, 39]. This is in contrast to our findings where CRC risk was decreased in aspirin users with wild-type genotype compared to non-users. Analysis for the interaction between the *UGT1A6* SNP variant allele, any NSAID (including aspirin) use and CRC risk in the current study revealed no difference in the direction of association compared to the interaction involving aspirin only use (data not presented). Whilst testing for the source of the contradiction between these observations was beyond the remit of the current study, it is plausible to attribute the differences to the distinction between the key genetic and epigenetic molecular factors between adenoma and carcinoma that may make carcinoma cells more sensitive to aspirin intervention [40]. One of the key changes observed during adenoma to carcinoma progression is change in gene expression [41, 42]. One of the genes downregulated during the adenoma-carcinoma progression is *UGT1A6*. As the wild-type allele has lower metabolic activity compared to the variant allele, participants with wild-type genotype are likely to retain active metabolites of aspirin longer than the mutant allele carriers; hence, deriving greater chemopreventive benefit. Moreover, this is in keeping with observations in chemoprevention trials CAPP1 and CAPP2 where the chemopreventive effect of aspirin was restricted to colorectal cancer incidence with apparently limited impact on colorectal adenoma development [43, 44]. Additionally, the site-specific interaction of *UGT1A6* SNPs with colon cancer has been observed previously, albeit without statistical significance due to small study size consisting of only 422 cases and 481 population controls [45].

Interestingly, SNP rs11694911, which is located downstream to the *ODC1* gene has previously been shown to be associated with increased risk of colorectal adenoma [16]. In contrast, we observed an association between the SNP variant allele and 16% reduction in colorectal cancer risk. Sample sizes in both studies were reasonable and to date, no functional evidence about the SNP is available. Among individuals with the wild type rs2965667 SNP at *MGST1* locus, we found decreased CRC risk among aspirin users; this finding is consistent in direction but not as strong as the findings from the GWAS study of Nan *et al*. 2015 where the study involved over 8000 cases and controls. However, for individuals carrying the variant allele, that study showed significantly increased risk of CRC among NSAID users, a result inconsistent with our observations. The current study didn’t observe evidence of interaction between variant alleles of SNPs in previously reported genes- *ALOX15*, *IL16*, *MDR1*, *NFkB*, *PTGS1 and PTGS2-* with aspirin only use and CRC or colon cancer risk. This could in part be attributed to the differences in the choice of drug definition for carrying out the interaction test. Previous studies carried out GxE interaction test with any NSAID use (including aspirin) and CRC risk. The broad range of NSAIDs used to carry out the test includes drugs with different pharmacokinetic and pharmacodynamics mechanisms thus precluding the assessment of the true source of interaction.

An *ad hoc* power calculation was performed in Quanto V1.2.4 (http://biostats.usc.edu/Quanto.html) using the outcomes observed in the current study to assess the number of cases and controls needed to carry out an interaction test between *UGT1A6* SNP, aspirin only use and colorectal cancer risk with two-sided *P* value of 0.002 (corrected for multiple tests) and 80% power. Assuming an unmatched case-control (1:1 ratio) study design, population CRC risk (P_o_) of 6%, SNP minor allele frequency of 31% with additive mode of inheritance, genetic effect (R_G_) of 5%, environmental effect (aspirin only users; R_E_) of 26% and population prevalence of aspirin users (P_E_) of 20%: we would require 7057 cases and controls to observe an GxE interaction effect (R_GE_) of 20%. This highlights that the current study with a combined total of 3325 cases and 2262 controls had limited statistical power for GxE interaction. Whilst we did not adjust analysis for multiple test correction, hence increasing the probability of false positive results, we did observe results for some of the SNPs that are congruent with previously published studies where correction for multiple testing was applied. Even though these results are consistent with ours, all positive results should be regarded as hypothesis-generating and should be investigated in other independent datasets.

Additionally, we could not investigate whether there was a dose-response relationship among aspirin users because dose information was not recorded in the NIH-CCFR dataset. Other NSAIDs were also used by participants in both studies but numbers precluded detailed examination of each. Compared with previous studies where analysis for interaction was carried out in all users of NSAIDs–and thus limiting identification of the true source of interaction as each NSAID affect different biological pathways–our study was restricted to interaction in aspirin-only users.

In summary, our results suggest variant alleles of the genes involved in aspirin pathways, *CYP2C9*, *ODC1* and *UGT1A6*, may be involved in the modification of CRC or colon cancer risk independently or in conjunction with aspirin use.

## Supporting information

S1 File Anonymised UK-CCSG raw dataset. (XLSX)

S2 File A detailed description of genotyping, quality assurance and control, and imputation. (DOCX)

S3 File Meta-analysis of genetic association studies checklist. (DOCX)

S1 Table Standardized definitions of common data elements between the UK-CCSG and NIH-CCFR datasets. + Smoking includes cigarettes, cigar and pipes. ^Alcohol includes beer, cider, wine, sherry, other fortified wine, sake, champagne and spirits.
(DOCX)

S2 Table List of SNPs from genes included in the study. (DOCX)

S3 Table List of SNPs genotyped across different platforms in UK-CCSG and NIH-CCFR datasets. (DOCX)

S4 Table Comparison of observed minor allele frequency of SNPs between UK-Colorectal Cancer Stud Group and NIH-Colon Cancer Study Registry. * Observed minor allele frequency (MAF) in controls was compared to the MAF reported for Phase I GBR and Phase I CEU population from 1000 Genomes database in UK-CCSG and NIH-CCFR datasets respectively using Fisher’s exact test. ^**+**^ Observed MAF in controls of the two datasets were compared using Fisher’s exact test. ^**A**^ rs16973225 and rs5277 were only genotyped in cases in the NIH-CCFR dataset.
(DOCX)

S5 Table Baseline epidemiological characteristics data distribution within the UK-CCSG and NIH-CCFR datasets. a, Total number of subjects calculated from only CCO, UQM and FHCRC study sites.
b, *P*-value calculated using Fisher’s exact test for categorical variables and Mann-Whitney U test for continuous variables.
LTRI = Lunenfeld-Tanenbaum Research Institute, Ontario; USC = University of Southern California; UoM = University of Melbourne; MC = Mayo Clinic; FHCRC = Fred Hutchinson Cancer Research Centre; UHI = University of Hawaii Cancer Center.
(DOCX)

S6 Table Association between SNP variant allele and colorectal cancer risk. *P-value for association adjusted for age, sex and study site.
CI, Confidence Interval
n, Number of subjects.
(DOCX)

S7 Table Meta-analysis of association between SNP variant allele and colorectal cancer risk. *P-value for association adjusted for age, sex and study site.
+*P*-value for Cochran’s Q-test for heterogeneity.
CI, Confidence Interval.
(DOCX)

S8 Table Association between SNP variant allele and site-specific colorectal cancer risk. +*P*-value for association adjusted for age, sex and study site.
CI, Confidence Interval
n, Number of subjects.
(DOCX)

S9 Table Meta-analysis of association between SNP variant allele and colon cancer risk. *P-value for association adjusted for age, sex and study site.
+*P*-value for Cochran’s Q-test for heterogeneity.
CI, Confidence Interval.
(DOCX)

S10 Table Meta-analysis of association between SNP variant allele and rectal cancer risk. *P-value for association adjusted for age, sex and study site.
+*P*-value for Cochran’s Q-test for heterogeneity.
CI, Confidence Interval.
(DOCX)

S11 Table Interaction between SNP variant allele, aspirin only use and colorectal cancer. +*P*-value for association adjusted for age, sex and study site.
*P-value for interaction between SNP variant allele, aspirin use and colorectal cancer risk calculated using Likelihood ratio test. *P-*value is adjusted for age, sex and study site.
OR, Odds Ratio
CI, Confidence Interval
n, Number of subjects.
(DOCX)

S12 Table Meta-analysis of interaction between SNP variant allele, aspirin only use and colorectal cancer. +*P*-value for association adjusted for age, sex and study site.
*P-value for interaction between SNP variant allele, aspirin use and colorectal cancer risk calculated using Likelihood ratio test. *P-*value is adjusted for age, sex and study site.
~*P-*value for Cochran’s Q-test for heterogeneity
OR, Odds Ratio
CI, Confidence Interval.
(DOCX)

S13 Table Sensitivity analysis- meta-analysis of interaction between SNP variant allele, aspirin only use and colorectal cancer, in cases and controls with age more than 50 years. +*P*-value for association adjusted for age, sex and study site.
*P-value for interaction between SNP variant allele, aspirin use and colorectal cancer risk calculated using Likelihood ratio test. *P-*value is adjusted for age, sex and study site.
~*P-*value for Cochran’s Q-test for heterogeneity
OR, Odds Ratio
CI, Confidence Interval.
(DOCX)

S1 Fig Linkage disequilibrium (R^2^) heat maps for SNPs in the UK-Colorectal Cancer Study Group dataset. (DOCX)

S2 Fig Linkage disequilibrium (R^2^) heat maps for SNPs in the NIH-Colon Cancer Family Registry dataset. (DOCX)
