# Supplementary material for: Interaction between polymorphisms in aspirin metabolic pathways, regular aspirin use and colorectal cancer risk: A case-control study in unselected white European populations
Source: PLoS One. 2018 Feb 9;13(2):e0192223. doi: 10.1371/journal.pone.0192223 (PMC5806861; doi:10.1371/journal.pone.0192223)
Supplement: S2 File — (DOCX) [file pone.0192223.s002.docx]

S2 File: A detailed description of genotyping, quality assurance and control, and imputation

### UK-Colorectal Cancer Study Group

Cases and controls provided a venous blood sample at the time of the interview, or shortly after, which was stored in an EDTA vacutainer tube at -20°C. Genomic DNA was extracted from leukocytes using Nucleon BACC2 Genomic DNA extraction kit at the study sites (Gen-Probe Life Sciences, Manchester, UK). An aliquot of genomic DNA sample was sent to either Tepnel Pharma Services Ltd. (Manchester, UK) or Wellcome Trust Clinical Research Facility (Edinburgh, UK) for genotyping >240,000 SNPs using the Illumina HumanExome BeadChip array V1.1 (Illumina, San Diego, USA). SNPs were automatically called using the Illumina GenomeStudio data analysis software (Illumina, San Diego, USA). Overall, genotyping call rate for the samples was 97.21%. A 100% match for the genotype call was observed for 64 sample replicates that were genotyped at both facilities.

SNPs with a minor allele frequency (MAF) of >3% that were absent on the Illumina HumanExome BeadChip array v1.1 were genotyped using TaqMan drug metabolizing genotyping assay for allelic discrimination (Applied Biosystems, Paisley, UK) at St. James’s Hospital, Leeds. Primer design, optimization and genotyping assay was carried out by the UK-CCSG study group and the genotype data was made available for analysis. To carry out the assay, genomic DNA samples were robotically replica-plated in a series of 96-well daughter plates. The end-point fluorescence was read using an ABI PRISM 7700 sequence detection system (Applied Biosystems, Paisley, UK) and analyzed using Sequence Detector Software V1.7a. For quality control, each 96 well plate included previously analyzed samples representative of each genotype where the genotype had been verified by sequencing along with multiple no-template control samples. In addition, 1% of the samples were selected at random for repeat analysis. Overall, the failure rate was <2%.

### NIH-Colon Cancer Family Registry- Genotyping information

Peripheral blood was collected using standardized procedures from cases and controls (1). DNA samples were genotyped at University of Southern California on 3 separate platforms: Illumina 1M, Illumina 1M-Duo and Illumina HumanOmni1 arrays (Illumina, San Diego, USA). SNPs were automatically called using the Illumina GenomeStudio data analysis software (Illumina, San Diego, USA). Genome wide SNP data was provided by Prof. Graham Casey and was stored on a server at St. James’s Hospital, Leeds. SNPs absent on the array were imputed by using proxy SNPs (linkage disequilibrium R^2^=1.0) from HapMap II CEU population. SNP rs20417 was previously imputed at USC and thus the imputed genotype data was provided by them for analysis. SNP call rate and QC information has been described previously (2).

### References:

1. Newcomb PA, Baron J, Cotterchio M, Gallinger S, Grove J, Haile R, et al. Colon Cancer Family Registry: an international resource for studies of the genetic epidemiology of colon cancer. Cancer epidemiology, biomarkers & prevention : a publication of the American Association for Cancer Research, cosponsored by the American Society of Preventive Oncology. 2007;16(11):2331-43.

2. Figueiredo JC, Lewinger JP, Song C, Campbell PT, Conti DV, Edlund CK, et al. Genotype-environment interactions in microsatellite stable/microsatellite instability-low colorectal cancer: results from a genome-wide association study. Cancer epidemiology, biomarkers & prevention : a publication of the American Association for Cancer Research, cosponsored by the American Society of Preventive Oncology. 2011;20(5):758-66.
