# Supplementary material for: Interaction between polymorphisms in aspirin metabolic pathways, regular aspirin use and colorectal cancer risk: A case-control study in unselected white European populations
Source: PLoS One. 2018 Feb 9;13(2):e0192223. doi: 10.1371/journal.pone.0192223 (PMC5806861; doi:10.1371/journal.pone.0192223)
Supplement: S3 File — (DOCX) [file pone.0192223.s003.docx]

**Meta-analysis on Genetic Association Studies Checklist | PLOS ONE**

|  | Item | Section name and paragraph number within manuscript |
| --- | --- | --- |
|  | **Introduction** |  |
| 1 | Provide a detailed justification for the polymorphism studied; if a single polymorphism was analyzed, give details as to why others were not included in the meta-analysis. | Introduction, paragraph 2, lines 81-93 |
| 2 | Provide a detailed justification for the population(s) and clinical condition studied. | Introduction, paragraph 1, lines 64-70 |
|  | **Methods** |  |
| 3 | Provide full details of the search strategy employed; outline the full electronic search strategy –specific combination of keywords and any limits applied- for at least one database. Specify whether synonyms of polymorphisms/genes (e.g. SNP number) were searched. | SNP selection and genotyping, paragraph 1, lines 153-164 |
| 4 | Report full details on the inclusion and exclusion criteria applied for selecting studies.  *Please list the excluded articles and the reasons for exclusion of each article in a supplementary file.* | SNP selection and genotyping, paragraph 1 and 2, lines 153-175 |
| 5 | Provide details on how the quality of the studies included in the analyses was assessed. | SNP selection and genotyping, paragraph 1, lines 160-164 |
| 6 | Describe steps taken to contact study authors to identify additional studies and to request missing data. | N/A |
| 7 | Describe how environmental effects were adjusted for, if this adjustment was not conducted, outline the reasons for this. | Statistical analysis, paragraph 1, lines 189-196.  Statistical analysis, paragraph 2, lines 1197-201. |
| 8 | Describe the methods of handling heterogeneity/between-study variance. | Statistical analysis, paragraph 3, lines 215-217 |
| 9 | Describe how the Hardy-Weinberg equilibrium and linkage disequilibrium were assessed. | SNP selection and genotyping, paragraph 4, lines 183-185 |
| 10 | Describe and justify the choice of model for the analyses (per-allele vs per-genotype vs genetic model-free, random effects vs fixed effects). | Statistical analysis, paragraph 2, lines 200-201 and lines 207-210.  Statistical analysis, paragraph 3, lines 211-217. |
| 11 | Describe whether a sensitivity analysis has been completed. | Yes. Sensitivity Analysis, lines 315-326. |
| 12 | Describe whether an assessment of the effects of population stratification has been conducted. | N/A |
| 13 | Describe whether study-specific results have been assessed and if so the reasons for this (e.g. forest plot). | Statistical analysis, paragraph 3, lines 211-219 |
|  | **Results** |  |
| 14 | Include flow diagram for the studies included in the meta-analysis as the first figure for the manuscript | N/A. As only 2 datasets are included. Supplementary S2 Table contains list of SNPs investigated in the analysis. |
| 15 | Report details on allele/genotype prevalence. | See S4 Table. |
| 16 | Report the effect size estimates and p values for each analysis. | See S5-S13 Tables. |
|  | **Discussion** |  |
| 17 | Discuss the limitations of the meta-analysis, including genotyping errors/bias and publication bias. | Paragraph 5 and 6, lines 405-427. |
| 18 | If the meta-analysis identifies an association within a subgroup of the population studied but not another, discuss the implications of these results, and if applicable the possibility of subgroup-specific publication bias. | Paragraph 1 to 3, lines 329-387. |
| 19 | Discuss the suitability of the sample size employed to the research question and the power of the study. | Paragraph 5, lines 405-420. |
