# Supplementary material for: Interaction between polymorphisms in aspirin metabolic pathways, regular aspirin use and colorectal cancer risk: A case-control study in unselected white European populations
Source: PLoS One. 2018 Feb 9;13(2):e0192223. doi: 10.1371/journal.pone.0192223 (PMC5806861; doi:10.1371/journal.pone.0192223)
Supplement: S1 Table — + Smoking includes cigarettes, cigar and pipes. ^Alcohol includes beer, cider, wine, sherry, other fortified wine, sake, champagne and spirits. (DOCX) [file pone.0192223.s004.docx]

S1 Table: Standardized definitions of common data elements between the UK-CCSG and NIH-CCFR datasets.

| **Common Data Element** | **UK-CCSG definition** | **NIH-CCFR definition** | **Standardized definition** | **Coding terms** |
| --- | --- | --- | --- | --- |
| **Age** | Cases: age at cancer diagnosis,  Controls: age at interview | Cases: age at cancer diagnosis,  Controls: age at interview | Cases: age at cancer diagnosis,  Controls: age at interview | Continuous variable |
| **Sex** | Male coded as 0, female coded as 1 | Male coded as 1, female coded as 2 | Male coded as 0, female coded as 1 | 0 for male, 1 for female |
| **BMI** | BMI at 1 year before interview | BMI at 2 years before the interview | Keep respective study definitions | Continuous variable |
| **BMI at 20 years of age** | BMI at 20 years | BMI at 20 years | BMI at 20 years | Continuous variable |
| **Regular Smoking+** | Smoked 1 cigarette a day for 1 year | Smoked 1 cigarette a day for 3 months | Smoked 1 cigarette a day for 3 months | 0 for non-smoker, 1 for smoker |
| **Smoking before diagnosis+** | Regular smoking 1 year before diagnosis | Regular smoking 1 year before diagnosis | Regular smoking 1 year before diagnosis | 0 for non-smoker, 1 for smoker |
| **Alcohol intake^** | Alcohol intake of ≥1 drink a week at the age 40 years | Alcohol intake of ≥1 drink a week for at least 6 months at the age 30-40years | Alcohol intake of ≥1 drink a week at the age 30-40 years | 0 for non-drinker, 1 for drinker |
| **Alcohol intake unit^** | Alcohol units based on intake quantity | Alcohol units based on intake quantity | Alcohol units based on intake quantity | Continuous variable |
| **Exercise** | Hours spent on activities in a week a year ago from interview | Hours spent on activities in a week in 30s and 40s | Keep respective study definitions | Continuous variable |
| **Aspirin use** | Regular aspirin use for 3 months or longer | At least twice a week for more than a month | Keep respective study definitions | 0 for non-user, 1 for user |
| **NSAIDs use** | Regular NSAIDs use for 3 months or longer | At least twice a week for more than a month | Keep respective study definitions | 0 for non-user, 1 for user |
| **Calorie intake** | Based on average diet 1 year before the interview | Based on average diet 2 years before the interview | Keep respective study definitions | Continuous variable |
| **Family risk** | First degree and/or second degree relative with colorectal cancer | First degree and/or second degree relative with colorectal cancer | First degree and/or second degree relative with colorectal cancer | 0 for no, 1 for yes |

+ Smoking includes cigarettes, cigar and pipes

^Alcohol includes beer, cider, wine, sherry, other fortified wine, sake, champagne and spirits
