# Supplementary material for: Interaction between polymorphisms in aspirin metabolic pathways, regular aspirin use and colorectal cancer risk: A case-control study in unselected white European populations
Source: PLoS One. 2018 Feb 9;13(2):e0192223. doi: 10.1371/journal.pone.0192223 (PMC5806861; doi:10.1371/journal.pone.0192223)
Supplement: S2 Table — (DOCX) [file pone.0192223.s005.docx]

S2 Table: List of SNPs from genes included in the study.

| **Number** | **Gene Name** | **SNP ID** | **Association or interaction with colorectal adenoma, carcinoma and NSAID use** | **Study Reference** |
| --- | --- | --- | --- | --- |
| **1** | *ALOX15* | rs2619112 | Interaction with NSAID use and rectal cancer (*P*=0.01) | [1] |
| **2** | *CES2* | rs140461033 | Metabolize aspirin in small intestine. No association or interaction test conducted to date. SNPs selected based on their presence on the Illumina platforms. | [2] |
|  |  | rs44410046 |  |  |
|  |  | rs201103548 |  |  |
|  |  | rs28382815 |  |  |
|  |  | rs148026549 |  |  |
|  |  | rs145407778 |  |  |
|  |  | rs10852434 |  |  |
|  |  | rs14792040 |  |  |
|  |  | rs141625476 |  |  |
|  |  | rs147070911 |  |  |
|  |  | rs150408050 |  |  |
|  |  | rs147694237 |  |  |
| **3** | *CYP2C9* | rs1057910 | rs1799853 association with CRC risk (OR= 0.92, 95% CI=0.86-0.98, *P=*0.01) and association with adenoma risk (OR= 1.39, 95% CI=1.07-1.81, *P=*0.01) | [3] |
|  |  | rs1799853 |  |  |
| **4** | *IkBkB* | rs10958713 | Association with colon cancer risk (rs10958713 OR=0.62, 95% CI=0.42-0.92, *P*=0.03; rs5029748 OR=0.72, 95% CI=0.56-0.91, *P*=0.08)  Interaction with NSAID use and colon cancer risk (*P*=0.05 for rs11986055 and *P=*0.01 for rs6474387) | [4] |
|  |  | rs11986055 |  |  |
|  |  | rs5029748 |  |  |
|  |  | rs6474387 |  |  |
| **5** | *IL16* | rs12910333 | Interaction with NSAID use and colorectal cancer risk (*P*=8.2 x 10^-9^) | [5] |
|  |  | rs16973225 |  | [5] |
| **6** | *CCAT2* | rs6983267 | Association with colorectal cancer risk (OR=0.83, 95% CI= 0.74-0.94, *P*=0.004) | [6] |
|  | *Intergenic* 20p12 | rs961253 | Association with colorectal cancer risk (OR=1.10, 95% CI=1.04-1.17, *P=* 7.74 x 10^-4^) | Unpublished data from UK-CCSG group |
| **7** | *MDR1* | rs1045642 | Association with aspirin resistance (OR=2.47, 95% CI= 1.90-4.63, *P*<0.001) | [7] |
| **8** | *NCF4* | rs5995355 | Association with colorectal cancer risk (OR=2.43, 95% CI= 1.73-3.39, *P*<0.0001) | [8] |
| **9** | *MGST1* | rs2965667 | Interaction with NSAID use and colorectal cancer risk (*P*= 4.6 x 10^-9^) | [5] |
| **10** | *NFkB* | rs230490 | Interaction with NSAID use and colorectal cancer risk (*P*= 0.01) | [4] |
| **11** | *ODC1* | rs11694911 | Association with colorectal adenoma recurrence risk (RR= 1.29, 95% CI=1.08-1.53, *P*= 0.005) | [9] |
|  |  | rs28362380 | Interaction with aspirin use and colorectal adenoma recurrence risk (*P=* 0.03) | [9] |
|  |  | rs2302615 | Association with colorectal adenoma recurrence risk (RR= 0.43, 95% CI=0.16-1.15) | [9, 10] |
|  |  | rs2430420 | Association with colorectal adenoma recurrence risk (RR= 1.20, 95% CI=1.03-1.40, *P*= 0.02)  Interaction with aspirin use and colorectal adenoma recurrence risk (*P=* 0.05) | [9] |
| **12** | *PAFAH1B2* | rs4936367 | Metabolize aspirin in erythrocytes and plasma. No association or interaction test conducted to date. SNPs selected based on their presence on the Illumina platforms. | [11] |
|  |  | rs7112513 |  |  |
|  |  | rs142710583 |  |  |
|  |  | rs185651296 |  |  |
|  |  | rs186808413 |  |  |
|  |  | rs78428934 |  |  |
| **13** | *PTGS1* | rs3842787 | Interaction with NSAID use and rectal cancer risk (*P*=0.05 in DALS dataset and *P*=0.46 in CCFR dataset) | [12] |
| **14** | *PTGS2* | rs20417 | Association with rectal cancer risk (OR= 1.95, 95% CI= 0.89-4.26, *P*=0.05 in DALS dataset and OR= 4.88, 95% CI= 1.54-15.45, *P*=0.01 in CCFR dataset)  Interaction with NSAID use and colorectal adenoma recurrence risk (*P*=0.07) | [12, 13] |
|  |  | rs5275 | Association with colon cancer risk (OR= 0.82, 95% CI=0.66-1.03, *P*= 0.20 in DALS dataset) | [12] |
|  |  | rs4648310 | Association with colorectal adenoma recurrence risk (RR=1.35, 95% CI=1.03-1.77) | [14] |
|  |  | rs2745557 | Interaction with aspirin use and rectal cancer risk (*P*=0.03 in DALS study)  Interaction with aspirin use and colon cancer risk (*P*=0.001 in CCFR study) | Unpublished data from NIH-CCFR group |
|  |  | rs689469 | Association with colorectal adenoma recurrence (HR= 3.00, 95% CI=1.16-7.76, *P*= 0.02) | [15] |
|  |  | rs5277 | Association with colorectal adenoma recurrence (OR=1.49, 95% CI= 1.00-2.23) | [14] |
| **15** | *UGT1A6* | rs2070959 | Association with colorectal cancer risk (*P*=0.04)  rs1105879 interaction with NSAID use and rectal cancer risk (*P*=0.02) | [16, 17] |
|  |  | rs1105879 |  |  |

**References:**

1. Kleinstein SE, Heath L, Makar KW, Poole EM, Seufert BL, Slattery ML, et al. Genetic variation in the lipoxygenase pathway and risk of colorectal neoplasia. Genes Chromosomes Cancer. 2013;52(5):437-49. Epub 2013/02/14. doi: 10.1002/gcc.22042. PubMed PMID: 23404351; PubMed Central PMCID: PMCPMC3698944.

2. Kubo T, Kim SR, Sai K, Saito Y, Nakajima T, Matsumoto K, et al. Functional characterization of three naturally occurring single nucleotide polymorphisms in the CES2 gene encoding carboxylesterase 2 (HCE-2). Drug metabolism and disposition: the biological fate of chemicals. 2005;33(10):1482-7. Epub 2005/07/22. doi: 10.1124/dmd.105.005587. PubMed PMID: 16033949.

3. Wang H, Ren L, He Y, Wei Y, Chen Z, Yang W, et al. Association between cytochrome P450 2C9 gene polymorphisms and colorectal cancer susceptibility: evidence from 16 case-control studies. Tumour biology : the journal of the International Society for Oncodevelopmental Biology and Medicine. 2014;35(5):4317-22. Epub 2014/01/15. doi: 10.1007/s13277-013-1566-5. PubMed PMID: 24414392.

4. Seufert BL, Poole EM, Whitton J, Xiao L, Makar KW, Campbell PT, et al. IkappaBKbeta and NFkappaB1, NSAID use and risk of colorectal cancer in the Colon Cancer Family Registry. Carcinogenesis. 2013;34(1):79-85. Epub 2012/09/25. doi: 10.1093/carcin/bgs296. PubMed PMID: 23002237; PubMed Central PMCID: PMCPMC3534188.

5. Nan H, Hutter CM, Lin Y, et al. Association of aspirin and nsaid use with risk of colorectal cancer according to genetic variants. Jama. 2015;313(11):1133-42. doi: 10.1001/jama.2015.1815.

6. Nan H, Morikawa T, Suuriniemi M, Imamura Y, Werner L, Kuchiba A, et al. Aspirin use, 8q24 single nucleotide polymorphism rs6983267, and colorectal cancer according to CTNNB1 alterations. J Natl Cancer Inst. 2013;105(24):1852-61. Epub 2013/12/10. doi: 10.1093/jnci/djt331. PubMed PMID: 24317174; PubMed Central PMCID: PMCPMC3866156.

7. Sharma V, Kaul S, Al-Hazzani A, Prabha TS, Rao PP, Dadheech S, et al. Association of C3435T multi drug resistance gene-1 polymorphism with aspirin resistance in ischemic stroke and its subtypes. Journal of the neurological sciences. 2012;315(1-2):72-6. Epub 2011/12/20. doi: 10.1016/j.jns.2011.11.027. PubMed PMID: 22177087.

8. Ryan BM, Zanetti KA, Robles AI, Schetter AJ, Goodman J, Hayes RB, et al. Germline variation in NCF4, an innate immunity gene, is associated with an increased risk of colorectal cancer. International journal of cancer Journal international du cancer. 2014;134(6):1399-407. Epub 2013/08/29. doi: 10.1002/ijc.28457. PubMed PMID: 23982929; PubMed Central PMCID: PMCPMC3947351.

9. Barry EL, Mott LA, Sandler RS, Ahnen DJ, Baron JA. Variants downstream of the ornithine decarboxylase gene influence risk of colorectal adenoma and aspirin chemoprevention. Cancer prevention research (Philadelphia, Pa). 2011;4(12):2072-82. Epub 2011/09/21. doi: 10.1158/1940-6207.capr-11-0300. PubMed PMID: 21930798; PubMed Central PMCID: PMCPMC3232321.

10. Hubner RA, Muir KR, Liu JF, Logan RF, Grainge MJ, Houlston RS. Ornithine decarboxylase G316A genotype is prognostic for colorectal adenoma recurrence and predicts efficacy of aspirin chemoprevention. Clinical cancer research : an official journal of the American Association for Cancer Research. 2008;14(8):2303-9. Epub 2008/04/17. doi: 10.1158/1078-0432.ccr-07-4599. PubMed PMID: 18413818.

11. Zhou G, Marathe GK, Willard B, McIntyre TM. Intracellular erythrocyte platelet-activating factor acetylhydrolase I inactivates aspirin in blood. The Journal of biological chemistry. 2011;286(40):34820-9. Epub 2011/08/17. doi: 10.1074/jbc.M111.267161. PubMed PMID: 21844189; PubMed Central PMCID: PMCPMC3186390.

12. Makar KW, Poole EM, Resler AJ, Seufert B, Curtin K, Kleinstein SE, et al. COX-1 (PTGS1) and COX-2 (PTGS2) polymorphisms, NSAID interactions, and risk of colon and rectal cancers in two independent populations. Cancer Causes Control. 2013;24(12):2059-75. Epub 2013/09/12. doi: 10.1007/s10552-013-0282-1. PubMed PMID: 24022467; PubMed Central PMCID: PMCPMC3913564.

13. Ulrich CM, Whitton J, Yu JH, Sibert J, Sparks R, Potter JD, et al. PTGS2 (COX-2) -765G > C promoter variant reduces risk of colorectal adenoma among nonusers of nonsteroidal anti-inflammatory drugs. Cancer epidemiology, biomarkers & prevention : a publication of the American Association for Cancer Research, cosponsored by the American Society of Preventive Oncology. 2005;14(3):616-9. Epub 2005/03/16. doi: 10.1158/1055-9965.epi-04-0510. PubMed PMID: 15767339.

14. Barry EL, Sansbury LB, Grau MV, Ali IU, Tsang S, Munroe DJ, et al. Cyclooxygenase-2 polymorphisms, aspirin treatment, and risk for colorectal adenoma recurrence--data from a randomized clinical trial. Cancer epidemiology, biomarkers & prevention : a publication of the American Association for Cancer Research, cosponsored by the American Society of Preventive Oncology. 2009;18(10):2726-33. Epub 2009/09/17. doi: 10.1158/1055-9965.epi-09-0363. PubMed PMID: 19755647; PubMed Central PMCID: PMCPMC2769932.

15. Kraus S, Hummler S, Toriola AT, Poole EM, Scherer D, Kotzmann J, et al. Impact of genetic polymorphisms on adenoma recurrence and toxicity in a COX2 inhibitor (celecoxib) trial: results from a pilot study. Pharmacogenetics and genomics. 2013;23(8):428-37. Epub 2013/06/20. doi: 10.1097/FPC.0b013e3283631784. PubMed PMID: 23778325.

16. Scherer D, Koepl LM, Poole EM, Balavarca Y, Xiao L, Baron JA, et al. Genetic variation in UGT genes modify the associations of NSAIDs with risk of colorectal cancer: colon cancer family registry. Genes Chromosomes Cancer. 2014;53(7):568-78. Epub 2014/03/29. doi: 10.1002/gcc.22167. PubMed PMID: 24677636.

17. Angstadt AY, Hartman TJ, Lesko SM, Muscat JE, Zhu J, Gallagher CJ, et al. The effect of UGT1A and UGT2B polymorphisms on colorectal cancer risk: Haplotype associations and gene–environment interactions. Genes, Chromosomes and Cancer. 2014;53(6):454-66. doi: 10.1002/gcc.22157.
