# Supplementary material for: Interaction between polymorphisms in aspirin metabolic pathways, regular aspirin use and colorectal cancer risk: A case-control study in unselected white European populations
Source: PLoS One. 2018 Feb 9;13(2):e0192223. doi: 10.1371/journal.pone.0192223 (PMC5806861; doi:10.1371/journal.pone.0192223)
Supplement: S3 Table — (DOCX) [file pone.0192223.s006.docx]

S3 Table: List of SNPs genotyped across different platforms in UK-CCSG and NIH-CCFR datasets.

| **SNP ID** | **Genotyping platforms for UK-CCSG dataset** | | **Genotyping platforms for NIH-CCFR dataset** | | |
| --- | --- | --- | --- | --- | --- |
|  | Illumina Human Exome Array BeadChip v1.1 | Taqman Allelic Discrimination Assay | Illumina Human 1M Array | Illumina Human 1M-Duo Array | Illumina Human Omni1 Array |
| **rs1045642** |  | ✓ | ✓ | ✓ | ✓ |
| **rs1057910** | ✓ |  | ✓ | ✓ |  |
| **rs1799853** | ✓ |  |  | ✓ | ✓ |
| **rs6983267** | ✓ |  | ✓ | ✓ | ✓ |
| **rs961253** | ✓ |  | ✓ |  |  |
| **rs11694911** |  | ✓ | ✓ | ✓ | ✓ |
| **rs28362380** |  | ✓ | ✓ | ✓ | Proxy SNP rs1405948 |
| **rs4936367** | ✓ |  | ✓ | ✓ | Proxy SNP rs1351452 |
| **rs7112513** | ✓ |  | ✓ | ✓ | Proxy SNP rs1351452 |
| **rs3842787** | ✓ |  | ✓ | ✓ | ✓ |
| **rs20417** |  | ✓ | Imputed | ✓ | ✓ |
| **rs2070959** | ✓ |  | ✓ | ✓ | ✓ |
| **rs1105879** | ✓ |  | ✓ | ✓ | ✓ |
| **rs2619112** |  | ✓ | ✓ | ✓ | ✓ |
| **rs10958713** |  | ✓ | ✓ | ✓ | ✓ |
| **rs11986055** |  | ✓ | ✓ | ✓ | ✓ |
| **rs12910333** |  | ✓ | ✓ | ✓ | ✓ |
| **rs5995355** |  | ✓ | Proxy SNP rs6000449 | Proxy SNP rs6000449 | Proxy SNP rs6000449 |
| **rs230490** |  | ✓ | Proxy SNP rs1313925 | Proxy SNP rs1313925 | ✓ |
| **rs5275** |  |  | ✓ | ✓ | ✓ |
| **rs4648310** |  | ✓ | ✓ | ✓ |  |
| **rs5029748** |  |  | ✓ | ✓ |  |
| **rs2745557** |  | ✓ | ✓ |  | ✓ |
| **rs6474387** |  |  |  | ✓ |  |
| **rs16973225** |  | ✓ |  |  | ✓ |
| **rs2302615** |  | ✓ |  |  |  |
| **rs2430420** |  | ✓ |  |  |  |
| **rs5277** |  | ✓ |  |  | ✓ |
| **rs2965667** |  | ✓ |  |  |  |
| **rs140461033** | ✓ |  |  |  |  |
| **rs144410046** | ✓ |  |  |  |  |
| **rs201103548** | ✓ |  |  |  |  |
| **rs28382815** | ✓ |  |  |  |  |
| **rs148026549** | ✓ |  |  |  |  |
| **rs145407778** | ✓ |  |  |  |  |
| **rs10852434** | ✓ |  |  |  |  |
| **rs147942040** | ✓ |  |  |  |  |
| **rs141625476** | ✓ |  |  |  |  |
| **rs147070911** | ✓ |  |  |  |  |
| **rs150408050** | ✓ |  |  |  |  |
| **rs147694237** | ✓ |  |  |  |  |
| **rs142710583** | ✓ |  |  |  |  |
| **rs185651296** | ✓ |  |  |  |  |
| **rs186808413** | ✓ |  |  |  |  |
| **rs78428934** | ✓ |  |  |  |  |
