# Supplementary material for: Interaction between polymorphisms in aspirin metabolic pathways, regular aspirin use and colorectal cancer risk: A case-control study in unselected white European populations
Source: PLoS One. 2018 Feb 9;13(2):e0192223. doi: 10.1371/journal.pone.0192223 (PMC5806861; doi:10.1371/journal.pone.0192223)
Supplement: S4 Table — * Observed minor allele frequency (MAF) in controls was compared to the MAF reported for Phase I GBR and Phase I CEU population from 1000 Genomes database in UK-CCSG and NIH-CCFR datasets respectively using Fisher’s exact test. + Observed MAF in controls of the two datasets were compared using Fisher’s exact test. A rs16973225 and rs5277 were only genotyped in cases in the NIH-CCFR dataset. (DOCX) [file pone.0192223.s007.docx]

S4 Table: Comparison of observed minor allele frequency of SNPs between UK-Colorectal Cancer Stud Group and NIH-Colon Cancer Study Registry.

| **SNP ID** | **UK-Colorectal Cancer Study Group** | | |  | **NIH-Colon Cancer Family Registry** | | | **MAF comparison between datasets+** |
| --- | --- | --- | --- | --- | --- | --- | --- | --- |
|  | Observed MAF | Hardy-Weinberg equilibrium (*P*-value) | MAF comparison (*P*-value)* |  | Observed MAF | Hardy-Weinberg equilibrium (*P*-value) | MAF comparison (*P*-value)* |  |
| **rs1045642** | 0.46 | 0.85 | 0.51 |  | 0.48 | 0.48 | 0.24 | 0.31 |
| **rs1057910** | 0.07 | 0.13 | 0.66 |  | 0.08 | 0.25 | 0.80 | 0.77 |
| **rs1799853** | 0.14 | 0.89 | **0.04** |  | 0.11 | 0.23 | **0.02** | 0.12 |
| **rs6983267** | 0.47 | 0.40 | 0.36 |  | 0.48 | 0.95 | 0.12 | 0.67 |
| **rs961253** | 0.37 | 0.13 | 0.24 |  | 0.35 | 0.26 | **0.02** | 0.08 |
| **rs11694911** | 0.12 | **0.04** | 0.79 |  | 0.12 | **0.02** | 0.25 | 0.91 |
| **rs28362380** | 0.09 | 0.69 | **0.04** |  | 0.09 | 0.30 | 0.67 | 0.89 |
| **rs4936367** | 0.10 | **0.03** | **0.002** |  | 0.11 | 0.87 | 0.65 | 0.07 |
| **rs7112513** | 0.10 | 0.60 | **0.01** |  | 0.11 | 0.87 | 0.66 | 0.59 |
| **rs3842787** | 0.09 | 0.66 | 0.76 |  | 0.07 | 0.65 | 0.59 | 0.19 |
| **rs20417** | 0.14 | 0.52 | 0.74 |  | 0.18 | 0.59 | 0.72 | 0.36 |
| **rs2070959** | 0.29 | **0.05** | 0.56 |  | 0.33 | 0.22 | 0.91 | 0.64 |
| **rs1105879** | 0.32 | 0.27 | 0.29 |  | 0.35 | 0.58 | 0.96 | 0.06 |
| **rs2619112** | 0.46 | 1.00 | 0.80 |  | 0.45 | **0.05** | 0.58 | 0.30 |
| **rs10958713** | 0.37 | 0.06 | 0.57 |  | 0.36 | 0.73 | 0.98 | 0.41 |
| **rs11986055** | 0.04 | 0.09 | 1.00 |  | 0.04 | 1.0 | 0.69 | 0.46 |
| **rs12910333** | 0.28 | 0.25 | 0.50 |  | 0.30 | 0.76 | 0.66 | 0.35 |
| **rs5995355** | 0.06 | 0.16 | 0.77 |  | 0.06 | 0.02 | 0.85 | 0.83 |
| **rs230490** | 0.44 | 0.73 | 0.66 |  | 0.41 | 0.43 | 0.53 | 0.24 |
| **rs5275** | - | - | - |  | 0.36 | 0.88 | 0.55 | - |
| **rs4648310** | 0.04 | **0.0003** | 0.90 |  | 0.04 | 0.63 | 0.65 | **0.03** |
| **rs5029748** | - | - | - |  | 0.25 | 0.45 | 0.27 | - |
| **rs2745557** | 0.18 | 0.91 | 0.53 |  | 0.17 | 0.10 | 0.14 | 0.34 |
| **rs6474387** | - | - | - |  | 0.06 | 1.0 | 0.49 | - |
| **rs16973225^A^** | 0.06 | 1.0 | 1.0 |  | - | - | - | - |
| **rs2302615** | 0.28 | 0.52 | 0.94 |  | - | - | - | - |
| **rs2430420** | 0.34 | 0.22 | **0.009** |  | - | - | - | - |
| **rs5277^A^** | 0.14 | 0.90 | 0.06 |  | - | - | - | - |
| **rs2965667** | 0.04 | 0.22 | 1.0 |  | - | - | - | - |
| **rs140461033** | **0.01** | **<0.0001** | 1.0 |  | - | - | - | - |
| **rs144410046** | **0.004** | **<0.0001** | 1.0 |  | - | - | - | - |
| **rs201103548** | **0.005** | **<0.0001** | - |  | - | - | - | - |
| **rs28382815** | **0.002** | **<0.0001** | **0.03** |  | - | - | - | - |
| **rs148026549** | **0.0005** | 0.99 | 1.0 |  | - | - | - | - |
| **rs145407778** | **0.002** | 0.96 | **0.006** |  | - | - | - | - |
| **rs10852434** | **0.00** | 1.0 | - |  | - | - | - | - |
| **rs147942040** | **0.005** | **<0.0001** | 1.0 |  | - | - | - | - |
| **rs141625476** | **0.004** | **<0.0001** | **0.001** |  | - | - | - | - |
| **rs147070911** | **0.005** | **<0.0001** | **0.001** |  | - | - | - | - |
| **rs150408050** | **0.004** | **<0.0001** | 1.0 |  | - | - | - | - |
| **rs147694237** | **0.005** | **<0.0001** | **0.001** |  | - | - | - | - |
| **rs142710583** | **0.004** | **<0.0001** | **0.001** |  | - | - | - | - |
| **rs185651296** | **0.003** | **<0.0001** | 0.30 |  | - | - | - | - |
| **rs186808413** | **0.015** | 0.64 | 1.0 |  | - | - | - | - |
| **rs78428934** | **0.002** | 0.96 | 1.0 |  | - | - | - | - |

* Observed minor allele frequency (MAF) in controls was compared to the MAF reported for Phase I GBR and Phase I CEU population from 1000 Genomes database in UK-CCSG and NIH-CCFR datasets respectively using Fisher’s exact test.

^+^ Observed MAF in controls of the two datasets were compared using Fisher’s exact test.

^A^ rs16973225 and rs5277 were only genotyped in cases in the NIH-CCFR dataset.
