# Supplementary material for: Interaction between polymorphisms in aspirin metabolic pathways, regular aspirin use and colorectal cancer risk: A case-control study in unselected white European populations
Source: PLoS One. 2018 Feb 9;13(2):e0192223. doi: 10.1371/journal.pone.0192223 (PMC5806861; doi:10.1371/journal.pone.0192223)
Supplement: S5 Table — a, Total number of subjects calculated from only CCO, UQM and FHCRC study sites. b, P-value calculated using Fisher’s exact test for categorical variables and Mann-Whitney U test for continuous variables. LTRI = Lunenfeld-Tanenbaum Research Institute, Ontario; USC = University of Southern California; UoM = University of Melbourne; MC = Mayo Clinic; FHCRC = Fred Hutchinson Cancer Research Centre; UHI = University of Hawaii Cancer Center. (DOCX) [file pone.0192223.s008.docx]

S5 Table: Baseline epidemiological characteristics data distribution within the UK-CCSG and NIH-CCFR datasets.

| **Baseline characteristics** | **UK-Colorectal Cancer Study Group** | | | |  | **NIH-Colon Cancer Family Registry** | | | | | | | ***P*-value^b^** |
| --- | --- | --- | --- | --- | --- | --- | --- | --- | --- | --- | --- | --- | --- |
|  | Dundee | Leeds | York | **Total** |  | LTRI | USC | UoM | MC | FHCRC | UHI | **Total^a^** |  |
| **Sex, n (%)** |  |  |  |  |  |  |  |  |  |  |  |  |  |
| Male | 284 (54.7) | 1339 (54.9) | 136 (60.2) | **1759 (55.2)** |  | 523 (51.3) | 89 (43.2) | 267 (50.9) | 136 (44.7) | 414 (48.4) | - | **1204 (50.2)** | <0.001 |
| Female | 235 (45.3) | 1100 (45.1) | 90 (39.8) | **1425 (44.8)** |  | 497 (48.7) | 117 (56.8) | 258 (49.1) | 168 (55.3) | 442 (51.6) | - | **1197 (49.9)** |  |
| **Age at diagnosis in cases (in years), n (%)** |  |  |  |  |  |  |  |  |  |  |  |  |  |
| <50 | 6 (4.2) | 118 (7.1) | 8 (7.1) | **132 (6.9)** |  | 236 (44.6) | 129 (62.6) | 240 (70.8) | 251 (82.6) | 199 (36.4) | - | **675 (47.7)** | <0.001 |
| 51-60 | 23 (16.0) | 301 (18.2) | 23 (20.5) | **347 (18.2)** |  | 107 (20.2) | 24 (11.7) | 99 (29.2) | 20 (6.6) | 127 (23.2) | - | **333 (23.5)** |  |
| 61-70 | 59 (41.0) | 558 (33.8) | 34 (30.4) | **651 (34.1)** |  | 154 (29.1) | 35 (17.0) | - | 23 (7.6) | 154 (28.2) | - | **308 (21.8)** |  |
| 71-80 | 56 (38.9) | 570 (34.5) | 43 (38.4) | **669 (35.0)** |  | 32 (6.1) | 18 (8.7) | - | 10 (3.3) | 67 (12.3) | - | **99 (7.0)** |  |
| 81-90 | - | 103 (6.2) | 4 (3.6) | **107 (5.6)** |  | - | - | - | - | - | - | **-** |  |
| 91-100 | - | 3 (0.2) | - | **3 (0.2)** |  | - | - | - | - | - | - | **-** |  |
| **Age at interview in controls (in years), n (%)** |  |  |  |  |  |  |  |  |  |  |  |  |  |
| ≤50 | 11 (2.9) | 30 (3.8) | 10 (8.9) | **51 (4.0)** |  | 64 (13.0) | - | 102 (54.8) | - | 3 (1.0) |  | **169 (17.1)** | <0.001 |
| 51-60 | 68 (18.2) | 102 (13.0) | 21 (18.8) | **191 (15.0)** |  | 138 (28.1) | - | 84 (45.2) | - | 105 (34.0) |  | **327 (33.2)** |  |
| 61-70 | 158 (42.3) | 320 (40.7) | 37 (33.0) | **515 (40.5)** |  | 176 (35.9) | - | - | - | 133 (43.0) |  | **309 (31.3)** |  |
| 71-80 | 126 (33.7) | 311 (39.6) | 41 (36.6) | **478 (37.6)** |  | 113 (23.0) | - | - | - | 68 (22.0) |  | **181 (18.4)** |  |
| 81-90 | 11 (2.9) | 23 (2.9) | 3 (2.7) | **37 (2.9)** |  | - | - | - | - | - | - | **-** |  |
| 91-100 | - | - | - | **-** |  | - | - | - | - | - | - | **-** |  |
| **Family History of Cancer, n (%)** |  |  |  |  |  |  |  |  |  |  |  |  |  |
| Unaffected | 368 (77.0) | 1573 (74.2) | 140 (76.9) | **2081 (74.9)** |  | 645 (63.2) | 100 (48.5) | 309 (58.9) | 145 (47.7) | 641 (74.9) | - | **1595 (66.4)** | <0.001 |
| Affected second degree relatives | 29 (6.1) | 173 (8.2) | 10 (5.5) | **212 (7.6)** |  | 163 (16.0) | 9 (4.4) | 117 (22.3) | 67 (22.0) | 62 (7.2) | - | **342 (14.2)** |  |
| Affected first or first and second degree relatives | 81 (17.0) | 373 (17.6) | 32 (17.6) | **486 (17.5)** |  | 212 (20.8) | 97 (47.1) | 99 (18.9) | 92 (30.3) | 153 (17.9) | - | **464 (19.3)** |  |
| **Smoking, n (%)** |  |  |  |  |  |  |  |  |  |  |  |  |  |
| Never | 209 (40.9) | 976 (40.2) | 80 (37.7) | **1265 (40.2)** |  | 387 (38.0) | 93 (45.2) | 234 (44.7) | 139 (46.3) | 344 (40.2) | - | **965 (40.2)** | <0.001 |
| Stopped | 222 (43.4) | 1042 (42.9) | 101 (47.6) | **1365 (43.3)** |  | 527 (51.8) | 88 (42.7) | 209 (39.9) | 126 (42.0) | 416 (48.6) | - | **1152 (48.0)** |  |
| Current | 80 (15.7) | 410 (16.9) | 31 (14.6) | **521 (16.5)** |  | 104 (10.2) | 25 (12.1) | 81 (15.5) | 35 (11.7) | 96 (11.2) | - | **281 (11.7)** |  |
| **Any NSAID use, n (%)** |  |  |  |  |  |  |  |  |  |  |  |  |  |
| No | 370 (72.5) | 1732 (71.2) | 167 (78.4) | **2269 (71.9)** |  | 611 (60.6) | 102 (49.5) | 394 (75.3) | 156 (53.1) | 387 (45.4) | - | **1392 (58.4)** | <0.001 |
| Yes | 140 (27.5) | 701 (28.8) | 46 (21.6) | **887 (28.1)** |  | 398 (39.4) | 104 (50.5) | 129 (24.7) | 38 (46.9) | 465 (54.6) | - | **992 (41.6)** |  |
| **Aspirin only use, n (%)** |  |  |  |  |  |  |  |  |  |  |  |  |  |
| No | 370 (79.7) | 1732 (79.0) | 167 (86.5) | **2269 (79.6)** |  | 611 (71.4) | 102 (61.1) | 394 (88.0) | 156 (69.6) | 387 (58.7) | - | **1392 (70.9)** | <0.001 |
| Yes | 94 (20.3) | 460 (21.0) | 26 (13.5) | **580 (20.4)** |  | 245 (28.6) | 65 (38.9) | 54 (12.1) | 68 (30.4) | 272 (41.3) | - | **571 (29.1)** |  |
| **Primary Cancer Site, n (%)** |  |  |  |  |  |  |  |  |  |  |  |  |  |
| Colon | 82 (56.9) | 1085 (65.6) | 55 (48.7) | **1222 (64.0)** |  | 279 (65.8) | 131 (69.3) | 188 (58.4) | 189 (63.6) | 337 (62.6) | - | **804 (62.6)** | 0.45 |
| Rectum | 62 (43.1) | 568 (34.4) | 58 (51.3) | **688 (36.0)** |  | 145 (34.2) | 58 (30.7) | 136 (41.6) | 108 (36.4) | 201 (37.4) | - | **480 (37.4)** |  |
| **BMI at 20 years (kg/m^2^), mean (SD)** | 22.3 (2.5) | 22.3 (3.2) | 22.5 (3.4) | **22.3 (3.1)** |  | 22.4 (3.6) | 22.1 (3.1) | 22.8 (3.9) | 23.1 (4.0) | 22.6 (4.4) | - | **22.6 (4.0)** | 0.47 |
| **Physical Activity (Hours/ week), mean (SD)** | 25.4 (17.1) | 23.1 (13.4) | 26.1 (19.7) | **23.7 (14.6)** |  | 4.1 (7.1) | 8.7 (9.4) | 6.5 (7.2) | 10.9 (15.7) | 6.5 (8.8) | - | **5.4 (7.7)** | <0.0001 |
| **Alcohol (Units/ day), mean (SD)** | 1.7 (3.0) | 3.2 (5.0) | 2.2 (3.1) | **2.9 (4.6)** |  | 0 (0) | 1.6 (1.7) | 1.8 (2.9) | 2.0 (2.5) | 2.2 (3.6) | - | **2.0 (3.3)** | 0.88 |

a, Total number of subjects calculated from only CCO, UQM and FHCRC study sites.

b, *P*-value calculated using Fisher’s exact test for categorical variables and Mann-Whitney U test for continuous variables.

LTRI= Lunenfeld-Tanenbaum Research Institute, Ontario; USC= University of Southern California; UoM= University of Melbourne; MC= Mayo Clinic; FHCRC= Fred Hutchinson Cancer Research Centre; UHI= University of Hawaii Cancer Center.
