# Supplementary material for: Interaction between polymorphisms in aspirin metabolic pathways, regular aspirin use and colorectal cancer risk: A case-control study in unselected white European populations
Source: PLoS One. 2018 Feb 9;13(2):e0192223. doi: 10.1371/journal.pone.0192223 (PMC5806861; doi:10.1371/journal.pone.0192223)
Supplement: S6 Table — *P-value for association adjusted for age, sex and study site. CI, Confidence Interval n, Number of subjects. (DOCX) [file pone.0192223.s009.docx]

S6 Table: Association between SNP variant allele and colorectal cancer risk.

|  | | | **UK-Colorectal Cancer Study Group** | | | | |  | **NIH-Colon Cancer Family Registry** | | | | |
| --- | --- | --- | --- | --- | --- | --- | --- | --- | --- | --- | --- | --- | --- |
| **Gene name** | **SNP ID** | **Copies of rare allele** | **Controls, n (%)** | **Cases, n (%)** | **Odds Ratio** | **95% CI** | ***P*-value*** |  | **Controls, n (%)** | **Cases, n (%)** | **Odds Ratio** | **95% CI** | ***P*-value*** |
| ***MDR1*** | rs1045642 | 0 | 297 (29.2) | 457 (28.8) |  |  |  |  | 257 (26.1) | 383 (27.1) |  |  |  |
|  |  | 1 or 2 | 722 (70.9) | 1128 (71.2) | 1.01 | 0.84, 1.20 | 0.94 |  | 728 (73.9) | 1032 (72.9) | 0.95 | 0.79, 1.14 | 0.93 |
| ***CYP2C9*** | rs1057910 | 0 | 850 (86.8) | 1489 (87.8) |  |  |  |  | 841 (85.8) | 992 (86.5) |  |  |  |
|  |  | 1 or 2 | 129 (13.2) | 207 (12.2) | 0.91 | 0.72, 1.16 | 0.45 |  | 139 (14.2) | 155 (13.5) | 0.95 | 0.74, 1.21 | 0.80 |
|  | rs1799853 | 0 | 712 (74.3) | 1276 (78.0) |  |  |  |  | 141 (78.8) | 126 (79.3) |  |  |  |
|  |  | 1 or 2 | 245 (25.7) | 359 (22.0) | 0.81 | 0.67, 0.98 | **0.03** |  | 38 (21.2) | 33 (20.8) | 0.97 | 0.58, 1.64 | 0.83 |
| ***CCAT2*** | rs6983267 | 0 | 284 (28.9) | 523 (31) |  |  |  |  | 267 (27.1) | 436 (30.9) |  |  |  |
|  |  | 1 or 2 | 694 (71.1) | 1169 (69.1) | 0.92 | 0.77, 1.10 | 0.37 |  | 717 (72.9) | 976 (69.1) | 0.83 | 0.70, 1.00 | 0.06 |
| ***Intergenic* 20p12** | rs961253 | 0 | 401 (41.0) | 655 (38.5) |  |  |  |  | 406 (41.3) | 573 (40.5) |  |  |  |
|  |  | 1 or 2 | 579 (59.1) | 1045 (61.5) | 1.12 | 0.95, 1.32 | 0.16 |  | 578 (58.7) | 842 (59.5) | 1.03 | 0.87, 1.22 | 0.99 |
| ***ODC1*** | rs28362380 | 0 | 842 (83.4) | 1293 (82.8) |  |  |  |  | 822 (83.4) | 1149 (81.3) |  |  |  |
|  |  | 1 or 2 | 168 (16.7) | 269 (17.2) | 1.00 | 0.84, 1.29 | 0.97 |  | 164 (16.6) | 2665 (18.7) | 1.16 | 0.93, 1.43 | 0.21 |
|  | rs11694911 | 0 | 789 (77.6) | 1278 (80.7) |  |  |  |  | 772 (78.3) | 1135 (80.2) |  |  |  |
|  |  | 1 or 2 | 228 (22.4) | 306 (19.3) | 0.85 | 0.70, 1.04 | 0.10 |  | 214 (21.7) | 280 (19.8) | 0.89 | 0.73, 1.09 | 0.31 |
|  | rs2430420 | 0 | 453 (44.4) | 699 (44.5) |  |  |  |  | - | - | - | - | - |
|  |  | 1 or 2 | 565 (55.6) | 888 (55.5) | 1.03 | 0.87, 1.21 | 0.75 |  | - | - | - | - | - |
|  | rs2302615 | 0 | 501 (51.6) | 862 (56.5) |  |  |  |  | - | - | - | - | - |
|  |  | 1 or 2 | 471 (48.4) | 663 (43.5) | 0.85 | 0.72, 1.00 | **0.05** |  | - | - | - | - | - |
| ***PAFAH1B2*** | rs4936367 | 0 | 782 (80.8) | 1296 (79.3) |  |  |  |  | 786 (79.7) | 1125 (79.5) |  |  |  |
|  |  | 1 or 2 | 185 (19.2) | 337 (20.7) | 1.08 | 0.88, 1.33 | 0.45 |  | 200 (20.3) | 290 (20.5) | 1.01 | 0.83, 1.24 | 0.64 |
|  | rs7112513 | 0 | 792 (80.6) | 1342 (79.1) |  |  |  |  | 779 (79.4) | 1122 (79.5) |  |  |  |
|  |  | 1 or 2 | 190 (19.4) | 355 (20.9) | 1.09 | 0.89, 1.34 | 0.40 |  | 202 (20.6) | 289 (20.5) | 0.99 | 0.81, 1.22 | 0.51 |
| ***PTGS1*** | rs3842787 | 0 | 631 (82.8) | 976 (79.9) |  |  |  |  | 843 (85.9) | 1242 (88.0) |  |  |  |
|  |  | 1 or 2 | 132 (17.2) | 245 (20.1) | 1.11 | 0.87, 1.41 | 0.39 |  | 139 (14.2) | 169 (12.0) | 0.83 | 0.65, 1.05 | 0.37 |
| ***PTGS2*** | rs4648310 | 0 | 952 (93.8) | 1483 (93.8) |  |  |  |  | 917 (93.1) | 1085 (94.1) |  |  |  |
|  |  | 1 or 2 | 63 (6.2) | 99 (6.2) | 1.03 | 0.74, 1.45 | 0.86 |  | 68 (6.9) | 68 (5.9) | 0.85 | 0.6, 1.20 | 0.28 |
|  | rs20417 | 0 | 750 (73.2) | 1115 (70.6) |  |  |  |  | 672 (68.2) | 976 (69.0) |  |  |  |
|  |  | 1 or 2 | 276 (26.8) | 464 (29.4) | 1.13 | 0.95, 1.36 | 0.20 |  | 313 (31.8) | 438 (31.0) | 0.96 | 0.81, 1.15 | 0.80 |
|  | rs2745557 | 0 | 613 (67.4) | 1150 (70.6) |  |  |  |  | 558 (69.7) | 838 (66.9) |  |  |  |
|  |  | 1 or 2 | 296 (32.6) | 478 (29.4) | 0.87 | 0.73, 1.05 | 0.13 |  | 243 (30.3) | 414 (33.1) | 1.13 | 0.94, 1.37 | 0.11 |
|  | rs5277 | 0 | 760 (73.7) | 1125 (71.1) |  |  |  |  | - | - | - | - | - |
|  |  | 1 or 2 | 271 (26.3) | 458 (28.9) | 1.16 | 0.97, 1.39 | 0.11 |  | - | - | - | - | - |
|  | rs5275 | 0 | - | - | - | - | - |  | 385 (41.3) | 618 (45.3) |  |  |  |
|  |  | 1 or 2 | - | - | - | - | - |  | 547 (58.7) | 746 (54.7) | 0.85 | 0.72, 1.01 | 0.17 |
| ***UGT1A6*** | rs1105879 | 0 | 459 (47.1) | 765 (44.9) |  |  |  |  | 412 (41.8) | 594 (42.0) |  |  |  |
|  |  | 1 or 2 | 517 (52.9) | 936 (55.2) | 1.12 | 0.95, 1.32 | 0.17 |  | 573 (58.2) | 819 (58.0) | 0.99 | 0.84, 1.17 | 0.77 |
|  | rs2070959 | 0 | 498 (51) | 815 (48.0) |  |  |  |  | 433 (44.0) | 636 (45.0) |  |  |  |
|  |  | 1 or 2 | 479 (49.0) | 881 (52.0) | 1.16 | 0.98, 1.36 | 0.08 |  | 551 (56.0) | 779 (55.1) | 0.96 | 0.82, 1.13 | 0.90 |
| ***IL16*** | rs16973225 | 0 | 774 (87.6) | 1420 (89.0) |  |  |  |  | - | - | - | - | - |
|  |  | 1 or 2 | 109 (12.4) | 175 (11.0) | 0.84 | 0.65, 1.10 | 0.21 |  | - | - | - | - | - |
|  | rs12910333 | 0 | 456 (51.0) | 854 (53.7) |  |  |  |  | 482 (48.9) | 737 (52.1) |  |  |  |
|  |  | 1 or 2 | 438 (49.1) | 734 (46.3) | 0.92 | 0.77, 1.09 | 0.31 |  | 504 (51.1) | 677 (47.9) | 0.88 | 0.75, 1.03 | 0.27 |
| ***IKBKB*** | rs11986055 | 0 | 852 (93.3) | 1487 (92.2) |  |  |  |  | 914 (92.9) | 1305 (92.3) |  |  |  |
|  |  | 1 or 2 | 61 (6.7) | 126 (7.8) | 1.20 | 0.86, 1.66 | 0.28 |  | 70 (7.1) | 109 (7.7) | 1.09 | 0.80, 1.49 | 1.00 |
|  | rs10958713 | 0 | 374 (41.0) | 679 (42.0) |  |  |  |  | 406 (41.2) | 609 (43.0) |  |  |  |
|  |  | 1 or 2 | 537 (59.0) | 939 (58.0) | 0.97 | 0.82, 1.15 | 0.76 |  | 579 (58.8) | 806 (57.0) | 0.93 | 0.79, 1.09 | 0.58 |
|  | rs5029748 | 0 | - | - | - | - | - |  | 557 (56.6) | 643 (55.8) |  |  |  |
|  |  | 1 or 2 | - | - | - | - | - |  | 428 (43.5) | 510 (44.2) | 1.03 | 0.87, 1.23 | 0.60 |
|  | rs6474387 | 0 | - | - | - | - | - |  | 161 (87.0) | 146 (90.1) |  |  |  |
|  |  | 1 or 2 | - | - | - | - | - |  | 24 (13.0) | 16 (9.9) | 0.74 | 0.38, 1.44 | 0.24 |
| ***NCF4*** | rs5995355 | 0 | 791 (88.0) | 1408 (88.0) |  |  |  |  | 874 (88.6) | 1224 (86.6) |  |  |  |
|  |  | 1 or 2 | 109 (12.0) | 194 (12.1) | 1.08 | 0.83, 1.40 | 0.58 |  | 112 (11.4) | 190 (13.4) | 1.21 | 0.94, 1.55 | 0.18 |
| ***ALOX15*** | rs2619112 | 0 | 264 (29.4) | 434 (27.6) |  |  |  |  | 288 (29.2) | 390 (27.6) |  |  |  |
|  |  | 1 or 2 | 632 (70.6) | 1137 (72.4) | 1.07 | 0.89, 1.29 | 0.46 |  | 698 (70.8) | 1024 (72.4) | 1.08 | 0.90, 1.30 | 0.55 |
| ***NFKB*** | rs230490 | 0 | 289 (32.2) | 499 (31.2) |  |  |  |  | 335 (34.0) | 478 (33.8) |  |  |  |
|  |  | 1 or 2 | 610 (67.8) | 1096 (68.8) | 1.02 | 0.85, 1.22 | 0.85 |  | 651 (66.0) | 937 (66.2) | 1.01 | 0.85, 1.20 | 0.73 |
| ***MGST1*** | rs2965667 | 0 | 668 (93.2) | 1372 (92.8) |  |  |  |  | - | - | - | - | - |
|  |  | 1 or 2 | 49 (6.8) | 105 (7.2) | 1.02 | 0.71, 1.48 | 0.91 |  | - | - | - | - | - |
| ***IL23R*** | rs6683455 | 0 | - | - | - | - | - |  | 755 (77.3) | 867 (75.5) |  |  |  |
|  |  | 1 or 2 | - | - | - | - | - |  | 222 (22.7) | 282 (24.5) | 1.11 | 0.90, 1.35 | 0.53 |
| ***PGDH*** | rs7349744 | 0 | - | - | - | - | - |  | 101 (54.6) | 205 (48.2) |  |  |  |
|  |  | 1 or 2 | - | - | - | - | - |  | 84 (45.4) | 220 (51.8) | 1.29 | 0.91, 1.82 | 0.46 |
| ***FLAP*** | rs17239025 | 0 | - | - | - | - | - |  | 162 (87.6) | 153 (93.9) |  |  |  |
|  |  | 1 or 2 | - | - | - | - | - |  | 23 (12.4) | 10 (6.1) | 0.46 | 0.21, 1.00 | 0.14 |

**P*-value for association adjusted for age, sex and study site.

CI, Confidence Interval

n, Number of subjects
