# Supplementary material for: Interaction between polymorphisms in aspirin metabolic pathways, regular aspirin use and colorectal cancer risk: A case-control study in unselected white European populations
Source: PLoS One. 2018 Feb 9;13(2):e0192223. doi: 10.1371/journal.pone.0192223 (PMC5806861; doi:10.1371/journal.pone.0192223)
Supplement: S7 Table — *P-value for association adjusted for age, sex and study site. +P-value for Cochran’s Q-test for heterogeneity. CI, Confidence Interval. (DOCX) [file pone.0192223.s010.docx]

S7 Table: Meta-analysis of association between SNP variant allele and colorectal cancer risk.

| **Gene name** | **SNP ID** | **Meta-analysis Odds Ratio** | **95% CI** | ***P-*value*** | **I-squared (%)** | ***P-*value^+^** |
| --- | --- | --- | --- | --- | --- | --- |
| ***MDR1*** | rs1045642 | 1.02 | 0.93, 1.11 | 0.73 | 0 | 0.91 |
| ***CYP2C9*** | rs1057910 | 0.91 | 0.77, 1.09 | 0.3 | 0 | 0.85 |
|  | rs1799853 | 0.85 | 0.72, 1.01 | 0.06 | 0 | 0.74 |
| ***CCAT2*** | rs6983267 | 0.86 | 0.79, 0.94 | **0.001** | 0 | 0.4 |
| ***Intergenic* 20p12** | rs961253 | 1.07 | 0.98, 1.17 | 0.14 | 0 | 0.55 |
| ***ODC1*** | rs28362380 | 1.09 | 0.92, 1.28 | 0.32 | 12.5 | 0.29 |
|  | rs11694911 | 0.84 | 0.74, 0.96 | **0.01** | 0 | 0.58 |
|  | rs2430420 | 1.04 | 0.92, 1.18 | 0.53 | - | - |
|  | rs2302615 | 0.85 | 0.74, 0.98 | **0.02** | - | - |
| ***PAFAH1B2*** | rs4936367 | 1.03 | 0.088, 1.20 | 0.73 | 12.9 | 0.28 |
|  | rs7112513 | 1.02 | 0.86,1.20 | 0.82 | 31.4 | 0.23 |
| ***PTGS1*** | rs3842787 | 0.94 | 0.80, 1.12 | 0.52 | 0 | 0.45 |
| ***PTGS2*** | rs4648310 | 0.91 | 0.72, 1.14 | 0.46 | 0 | 0.4 |
|  | rs20417 | 1.06 | 0.88, 1.28 | 0.52 | 61.4 | 0.11 |
|  | rs2745557 | 1 | 0.81, 1.23 | 0.98 | 67.6 | 0.08 |
|  | rs5275 | 0.93 | 0.81,1.06 | 0.25 | - | - |
|  | rs5277 | 1.14 | 0.96, 1.34 | 0.14 | - | - |
| ***UGT1A6*** | rs1105879 | 1.04 | 0.95, 1.14 | 0.42 | 0 | 0.6 |
|  | rs2070959 | 1.04 | 0.95, 1.14 | 0.41 | 0 | 0.54 |
| ***IL16*** | rs12910333 | 0.95 | 0.87, 1.05 | 0.33 | 0 | 0.91 |
| ***IKBKB*** | rs11986055 | 1.07 | 0.85, 1.35 | 0.55 | 0 | 0.57 |
|  | rs10958713 | 0.94 | 0.86, 1.03 | 0.18 | 0 | 0.84 |
|  | rs5029748 | 1.05 | 0.90,1.21 | 0.54 | - | - |
|  | rs6474387 | 0.73 | 0.38,1.42 | 0.36 | - | - |
| ***NCF4*** | rs5995355 | 1.14 | 0.96,1.36 | 0.14 | 0 | 0.53 |
| ***ALOX15*** | rs2619112 | 1.09 | 1.00, 1.19 | 0.06 | 0 | 0.53 |
| ***NFKB*** | rs230490 | 1.03 | 0.95, 1.13 | 0.47 | 0 | 0.55 |
| ***MGST1*** | rs2965667 | 1.02 | 0.71,1.47 | 0.92 | - | - |
| ***IL23R*** | rs6683455 | 1.13 | 0.93,1.38 | 0.21 | - | - |
| ***PGDH*** | rs7349744 | 1.14 | 0.81,1.61 | 0.45 | - | - |
| ***FLAP*** | rs17239025 | 0.61 | 0.28,1.30 | 0.2 | - | - |

**P-*value for association adjusted for age, sex and study site.

+*P*-value for Cochran’s Q-test for heterogeneity.

CI, Confidence Interval
