# Supplementary material for: Interaction between polymorphisms in aspirin metabolic pathways, regular aspirin use and colorectal cancer risk: A case-control study in unselected white European populations
Source: PLoS One. 2018 Feb 9;13(2):e0192223. doi: 10.1371/journal.pone.0192223 (PMC5806861; doi:10.1371/journal.pone.0192223)
Supplement: S8 Table — +P-value for association adjusted for age, sex and study site. CI, Confidence Interval n, Number of subjects. (DOCX) [file pone.0192223.s011.docx]

S8 Table: Association between SNP variant allele and site-specific colorectal cancer risk.

|  | | | UK-Colorectal Cancer Study Group | | | | | |  | | NIH-Colon Cancer Family Registry | | | | | |
| --- | --- | --- | --- | --- | --- | --- | --- | --- | --- | --- | --- | --- | --- | --- | --- | --- |
| **Gene name** | **SNP ID** | **Copies of rare allele** | **Colon, n (%)** | **Rectum, n (%)** | **Odds Ratio** | **95% CI** | ***P*-value*** |  | | **Colon, n (%)** | | **Rectum, n (%)** | **Odds Ratio** | **95% CI** | ***P*-value*** |  |
| ***MDR1*** | rs1045642 | 0 | 303 (29.1) | 154 (28.3) |  |  |  |  | | 218 (27.1) | | 125 (26.0) |  |  |  |  |
|  |  | 1 or 2 | 737 (70.9) | 391 (71.7) | 1.04 | 0.83, 1.31 | 0.71 |  | | 586 (72.9) | | 355 (74.0) | 1.06 | 0.82, 1.37 | 0.53 |  |
| ***CYP2C9*** | rs1057910 | 0 | 992 (88.9) | 497 (85.7) |  |  |  |  | | 553 (86.3) | | 345 (86.3) |  |  |  |  |
|  |  | 1 or 2 | 124 (11.1) | 83 (14.3) | 1.27 | 0.94, 1.72 | 0.12 |  | | 88 (13.7) | | 55 (13.8) | 1 | 0.70, 1.44 | 0.86 |  |
|  | rs1799853 | 0 | 857 (79.8) | 419 (74.7) |  |  |  |  | | 73 (79.4) | | 50 (79.4) |  |  |  |  |
|  |  | 1 or 2 | 217 (20.2) | 142 (25.3) | 1.39 | 1.09, 1.78 | **0.008** |  | | 19 (20.7) | | 13 (20.6) | 1 | 0.45, 2.21 | 0.88 |  |
| ***CCAT2*** | rs6983267 | 0 | 346 (31.3) | 177 (30.4) |  |  |  |  | | 249 (31.0) | | 150 (31.4) |  |  |  |  |
|  |  | 1 or 2 | 763 (68.7) | 406 (69.6) | 1.06 | 0.85, 1.32 | 0.58 |  | | 554 (69.0) | | 328 (68.6) | 0.98 | 0.77, 1.25 | 0.77 |  |
| ***Intergenic* 20p12** | rs961253 | 0 | 433 (38.8) | 222 (38.0) |  |  |  |  | | 332 (41.3) | | 182 (37.9) |  |  |  |  |
|  |  | 1 or 2 | 682 (61.2) | 363 (62.1) | 1.04 | 0.84, 1.28 | 0.75 |  | | 472 (58.7) | | 298 (62.1) | 1.15 | 0.91, 1.45 | 0.29 |  |
| ***ODC1*** | rs28362380 | 0 | 862 (84.0) | 431 (80.4) |  |  |  |  | | 656 (81.7) | | 390 (81.3) |  |  |  |  |
|  |  | 1 or 2 | 164 (16.0) | 105 (19.6) | 1.32 | 1.00, 1.73 | **0.05** |  | | 147 (18.3) | | 90 (18.8) | 1.03 | 0.77, 1.38 | 0.83 |  |
|  | rs11694911 | 0 | 843 (81.3) | 435 (79.5) |  |  |  |  | | 632 (78.6) | | 392 (81.7) |  |  |  |  |
|  |  | 1 or 2 | 194 (18.7) | 112 (20.5) | 1.08 | 0.83, 1.41 | 0.56 |  | | 172 (21.4) | | 88 (18.3) | 0.82 | 0.62, 1.10 | 0.18 |  |
|  | rs2430420 | 0 | 449 (43.1) | 250 (46.0) |  |  |  |  | | - | | - | - | - | - |  |
|  |  | 1 or 2 | 594 (56.9) | 308 (54.0) | 0.86 | 0.70, 1.07 | 0.18 |  | | - | | - | - | - | - |  |
|  | rs2302615 | 0 | 583 (57.8) | 279 (53.0) |  |  |  |  | | - | | - | - | - | - |  |
|  |  | 1 or 2 | 416 (42.2) | 247 (47.0) | 1.23 | 0.99, 1.53 | 0.06 |  | | - | | - | - | - | - |  |
| ***PAFAH1B2*** | rs4936367 | 0 | 860 (80.3) | 436 (77.6) |  |  |  |  | | 643 (80.0) | | 377 (78.5) |  |  |  |  |
|  |  | 1 or 2 | 211 (19.7) | 126 (22.4) | 1.13 | 0.87, 1.45 | 0.36 |  | | 161 (20.0) | | 103 (21.5) | 1.09 | 0.83, 1.44 | 0.75 |  |
|  | rs7112513 | 0 | 892 (80.1) | 450 (77.1) |  |  |  |  | | 640 (79.9) | | 377 (78.7) |  |  |  |  |
|  |  | 1 or 2 | 221 (19.9) | 134 (23.0) | 1.15 | 0.90, 1.47 | 0.26 |  | | 161 (20.1) | | 102 (21.3) | 1.08 | 0.81, 1.42 | 0.81 |  |
| ***PTGS1*** | rs3842787 | 0 | 644 (80.6) | 332 (78.7) |  |  |  |  | | 704 (88.0) | | 423 (88.1) |  |  |  |  |
|  |  | 1 or 2 | 155 (19.5) | 90 (21.3) | 1.12 | 0.83, 1.50 | 0.47 |  | | 96 (12.0) | | 57 (11.9) | 0.99 | 0.70, 1.40 | 0.91 |  |
| ***PTGS2*** | rs4648310 | 0 | 975 (94.2) | 508 (92.9) |  |  |  |  | | 605 (93.9) | | 380 (94.5) |  |  |  |  |
|  |  | 1 or 2 | 60 (5.8) | 39 (7.1) | 1.22 | 0.80, 1.86 | 0.36 |  | | 39 (6.1) | | 22 (5.5) | 0.9 | 0.52, 1.54 | 0.78 |  |
|  | rs20417 | 0 | 729 (70.2) | 386 (71.4) |  |  |  |  | | 553 (68.8) | | 338 (70.4) |  |  |  |  |
|  |  | 1 or 2 | 309 (29.8) | 155 (28.7) | 0.96 | 0.76, 1.22 | 0.76 |  | | 251 (31.2) | | 142 (29.6) | 0.93 | 0.72, 1.18 | 0.41 |  |
|  | rs2745557 | 0 | 749 (70.0) | 401 (71.7) |  |  |  |  | | 485 (68.4) | | 273 (65.6) |  |  |  |  |
|  |  | 1 or 2 | 319 (30.0) | 159 (28.3) | 0.92 | 0.73, 1.15 | 0.48 |  | | 224 (31.6) | | 143 (34.4) | 1.13 | 0.88, 1.47 | 0.29 |  |
|  | rs5277 | 0 | 745 (71.7) | 380 (69.9) |  |  |  |  | | - | | - | - | - | - |  |
|  |  | 1 or 2 | 294 (28.3) | 164 (30.1) | 1.11 | 0.88, 1.39 | 0.40 |  | | - | | - | - | - | - |  |
|  | rs5275 | 0 | - | - | - | - | - |  | | 361 (46.3) | | 202 (44.0) |  |  |  |  |
|  |  | 1 or 2 | - | - | - | - | - |  | | 418 (53.7) | | 257 (56.0) | 1.1 | 0.87, 1.39 | 0.43 |  |
| ***UGT1A6*** | rs1105879 | 0 | 483 (43.1) | 282 (48.1) |  |  |  |  | | 322 (40.2) | | 208 (43.3) |  |  |  |  |
|  |  | 1 or 2 | 634 (56.8) | 302 (51.9) | 0.82 | 0.67, 1.01 | 0.06 |  | | 480 (59.9) | | 272 (56.7) | 0.88 | 0.70, 1.10 | 0.29 |  |
|  | rs2070959 | 0 | 519 (46.5) | 296 (50.7) |  |  |  |  | | 348 (43.3) | | 221 (46.0) |  |  |  |  |
|  |  | 1 or 2 | 595(53.5) | 286 (49.3) | 0.85 | 0.69, 1.04 | 0.11 |  | | 456 (56.7) | | 259 (54.0) | 0.89 | 0.71, 1.12 | 0.39 |  |
| ***IL16*** | rs16973225 | 0 | 939 (89.7) | 481 (87.6) |  |  |  |  | | - | | - | - | - | - |  |
|  |  | 1 or 2 | 108 (10.3) | 67 (12.4) | 1.26 | 0.91, 1.76 | 0.17 |  | | - | | - | - | - | - |  |
|  | rs12910333 | 0 | 561 (53.9) | 293 (53.3) |  |  |  |  | | 432 (53.8) | | 239 (49.8) |  |  |  |  |
|  |  | 1 or 2 | 479 (46.1) | 255 (46.7) | 1.02 | 0.83, 1.26 | 0.88 |  | | 371 (46.2) | | 241 (50.2) | 1.17 | 0.34, 1.47 | 0.14 |  |
| ***IKBKB*** | rs11986055 | 0 | 988 (92.8) | 499 (90.9) |  |  |  |  | | 737 (91.7) | | 445 (92.9) |  |  |  |  |
|  |  | 1 or 2 | 76 (7.2) | 50 (9.1) | 1.28 | 0.87, 1.87 | 0.21 |  | | 67 (8.3) | | 34 (7.1) | 0.84 | 0.55, 1.29 | 0.46 |  |
|  | rs10958713 | 0 | 456 (43.0) | 223 (40.0) |  |  |  |  | | 364 (45.3) | | 195 (40.6) |  |  |  |  |
|  |  | 1 or 2 | 604 (57.0) | 335 (60.0) | 1.18 | 0.96, 1.46 | 0.12 |  | | 440 (54.7) | | 285 (59.4) | 1.21 | 0.96, 1.52 | 0.07 |  |
|  | rs5029748 | 0 | - | - | - | - | - |  | | 370 (57.5) | | 216 (53.7) |  |  |  |  |
|  |  | 1 or 2 | - | - | - | - | - |  | | 274 (45.6) | | 186 (46.3) | 1.16 | 0.90, 1.49 | 0.14 |  |
|  | rs6474387 | 0 | - | - | - | - | - |  | | 83 (87.4) | | 59 (93.7) |  |  |  |  |
|  |  | 1 or 2 | - | - | - | - | - |  | | 12 (12.6) | | 4 (6.4) | 0.47 | 0.14, 1.53 | 0.21 |  |
| ***NCF4*** | rs5995355 | 0 | 928 (88.1) | 480 (87.6) |  |  |  |  | | 695 (86.6) | | 417 (86.9) |  |  |  |  |
|  |  | 1 or 2 | 125 (11.9) | 69 (12.4) | 1.09 | 0.79, 1.50 | 0.60 |  | | 108 (13.5) | | 63 (13.1) | 0.97 | 0.70, 1.36 | 0.80 |  |
| ***ALOX15*** | rs2619112 | 0 | 278 (27.0) | 156 (28.9) |  |  |  |  | | 221 (27.5) | | 133 (27.7) |  |  |  |  |
|  |  | 1 or 2 | 756 (73.0) | 381 (71.1) | 0.89 | 0.70, 1.13 | 0.33 |  | | 582 (72.5) | | 347 (72.3) | 0.99 | 0.77, 1.28 | 0.78 |  |
| ***NFKB*** | rs230490 | 0 | 320 (30.6) | 179 (32.4) |  |  |  |  | | 264 (32.8) | | 167 (34.8) |  |  |  |  |
|  |  | 1 or 2 | 725 (69.4) | 370 (67.6) | 0.91 | 0.72, 1.13 | 0.39 |  | | 540 (67.2) | | 313 (65.2) | 0.92 | 0.72, 1.16 | 0.42 |  |
| ***MGST1*** | rs2965667 | 0 | 893 (93.2) | 479 (91.9) |  |  |  |  | | - | | - | - | - | - |  |
|  |  | 1 or 2 | 64 (6.8) | 41 (8.1) | 1.21 | 0.80, 1.83 | 0.38 |  | | - | | - | - | - | - |  |
| ***IL23R*** | rs6683455 | 0 | - | - | - | - | - |  | | 487 (75.7) | | 289 (72.4) |  |  |  |  |
|  |  | 1 or 2 | - | - | - | - | - |  | | 156 (24.3) | | 110 (27.6) | 1.19 | 0.89, 1.58 | 0.24 |  |
| ***PGDH*** | rs7349744 | 0 | - | - | - | - | - |  | | 126 (49.4) | | 62 (43.7) |  |  |  |  |
|  |  | 1 or 2 | - | - | - | - | - |  | | 129 (50.6) | | 80 (56.3) | 1.26 | 0.83, 1.90 | 0.22 |  |
| ***FLAP*** | rs17239025 | 0 | - | - | - | - | - |  | | 90 (94.7) | | 59 (92.2) |  |  |  |  |
|  |  | 1 or 2 | - | - | - | - | - |  | | 5 (5.3) | | 5 (7.8) | 1.53 | 0.42, 5.50 | 0.32 |  |

+*P*-value for association adjusted for age, sex and study site.

CI, Confidence Interval

n, Number of subjects
