# Supplementary material for: Interaction between polymorphisms in aspirin metabolic pathways, regular aspirin use and colorectal cancer risk: A case-control study in unselected white European populations
Source: PLoS One. 2018 Feb 9;13(2):e0192223. doi: 10.1371/journal.pone.0192223 (PMC5806861; doi:10.1371/journal.pone.0192223)
Supplement: S9 Table — *P-value for association adjusted for age, sex and study site. +P-value for Cochran’s Q-test for heterogeneity. CI, Confidence Interval. (DOCX) [file pone.0192223.s012.docx]

S9 Table: Meta-analysis of association between SNP variant allele and colon cancer risk.

| **Gene name** | **SNP ID** | **Meta-analysis Odds Ratio** | **95% CI** | ***P-*value*** | **I-squared (%)** | ***P-*value^+^** |
| --- | --- | --- | --- | --- | --- | --- |
| ***MDR1*** | rs1045642 | 0.99 | 0.90, 1.09 | 0.84 | 0 | 0.89 |
| ***CYP2C9*** | rs1057910 | 0.88 | 0.73, 1.07 | 0.19 | 0 | 0.54 |
|  | rs1799853 | 0.79 | 0.65, 0.95 | **0.01** | 0 | 0.52 |
| ***CCAT2*** | rs6983267 | 0.86 | 0.78, 0.95 | **0.002** | 0 | 0.42 |
| ***Intergenic* 20p12** | rs961253 | 1.06 | 0.96, 1.17 | 0.26 | 6.2 | 0.3 |
| ***ODC1*** | rs28362380 | 1.03 | 0.82, 1.30 | 0.78 | 46.4 | 0.17 |
|  | rs11694911 | 0.87 | 0.73, 1.05 | 0.14 | 33.5 | 0.22 |
|  | rs2430420 | 1.07 | 0.94, 1.22 | 0.32 | - | - |
|  | rs2302615 | 0.80 | 0.69, 0.93 | **0.004** | - | - |
| ***PAFAH1B2*** | rs4936367 | 1.02 | 0.87, 1.19 | 0.85 | 0 | 0.4 |
|  | rs7112513 | 1.01 | 0.87, 1.18 | 0.92 | 0 | 0.36 |
| ***PTGS1*** | rs3842787 | 0.92 | 0.76, 1.11 | 0.39 | 0 | 0.76 |
| ***PTGS2*** | rs4648310 | 0.94 | 0.72, 1.21 | 0.61 | 0 | 0.46 |
|  | rs20417 | 1.08 | 0.89, 1.32 | 0.43 | 58.1 | 0.12 |
|  | rs2745557 | 0.97 | 0.85, 1.11 | 0.70 | 0 | 0.36 |
|  | rs5275 | 0.91 | 0.79, 1.06 | 0.22 | - | - |
|  | rs5277 | 1.09 | 0.91, 1.31 | 0.36 | - | - |
| ***UGT1A6*** | rs1105879 | 1.09 | 0.99, 1.21 | 0.09 | 0 | 0.66 |
|  | rs2070959 | 1.09 | 0.99, 1.21 | 0.09 | 0 | 0.54 |
| ***IL16*** | rs12910333 | 0.92 | 0.82, 1.02 | 0.12 | 0 | 0.73 |
| ***IKBKB*** | rs11986055 | 1.06 | 0.82, 1.36 | 0.67 | 0 | 0.9 |
|  | rs10958713 | 0.9 | 0.82, 0.99 | **0.04** | 0 | 0.98 |
|  | rs5029748 | 0.98 | 0.83, 1.16 | 0.80 | - | - |
|  | rs6474387 | 0.97 | 0.48, 1.95 | 0.92 | - | - |
| ***NCF4*** | rs5995355 | 1.14 | 0.94, 1.38 | 0.20 | 0 | 0.4 |
| ***ALOX15*** | rs2619112 | 1.11 | 1.00, 1.22 | **0.05** | 0 | 0.52 |
| ***NFKB*** | rs230490 | 1.06 | 0.96, 1.17 | 0.23 | 0 | 0.63 |
| ***MGST1*** | rs2965667 | 0.96 | 0.65,1.43 | 0.85 | - | - |
| ***IL23R*** | rs6683455 | 1.15 | 0.92,1.44 | 0.22 | - | - |
| ***PGDH*** | rs7349744 | 1.11 | 0.76,1.61 | 0.58 | - | - |
| ***FLAP*** | rs17239025 | 0.56 | 0.22,1.40 | 0.21 | - | - |

**P-*value for association adjusted for age, sex and study site.

+*P*-value for Cochran’s Q-test for heterogeneity.

CI, Confidence Interval
