# Supplementary material for: Interaction between polymorphisms in aspirin metabolic pathways, regular aspirin use and colorectal cancer risk: A case-control study in unselected white European populations
Source: PLoS One. 2018 Feb 9;13(2):e0192223. doi: 10.1371/journal.pone.0192223 (PMC5806861; doi:10.1371/journal.pone.0192223)
Supplement: S11 Table — +P-value for association adjusted for age, sex and study site. *P-value for interaction between SNP variant allele, aspirin use and colorectal cancer risk calculated using Likelihood ratio test. P-value is adjusted for age, sex and study site. OR, Odds Ratio CI, Confidence Interval n, Number of subjects. (DOCX) [file pone.0192223.s014.docx]

S11 Table: Interaction between SNP variant allele, aspirin only use and colorectal cancer.

|  | | | **UK-Colorectal Cancer Study Group** | | | | |  | **NIH-Colon Cancer Family Registry** | | | | |
| --- | --- | --- | --- | --- | --- | --- | --- | --- | --- | --- | --- | --- | --- |
| **Gene name** | **SNP ID** | **Copies of rare allele** | **Non-users** | | **Aspirin users** | | ***P*-value for interaction*** |  | **Non-users** | | **Aspirin users** | | ***P*-value for interaction*** |
|  |  |  | **OR (95% CI)** | ***P*-value+** | **OR (95% CI)** | ***P*-value+** |  |  | **OR (95% CI)** | ***P*-value+** | **OR (95% CI)** | ***P*-value+** |  |
| ***MDR1*** | rs1045642 | 0 | n=539 1 |  | n=139 0.77 (0.63, 0.95) | 0.01 |  |  | n=392 1 |  | n=138 0.88 (0.71, 1.10) | 0.27 |  |
|  |  | 1 or 2 | n=1309 1.00 (0.82, 1.25) | 0.99 | n=348 0.91 (0.82, 1.00) | 0.05 | 0.49 |  | n=999 1.02 (0.79, 1.31) | 0.90 | n=433 0.94 (0.84, 1.04) | 0.24 | 1.00 |
| ***CYP2C9*** | rs1057910 | 0 | n=1682 1 |  | n=434 0.84 (0.76, 0.95) | 0.004 |  |  | n=1082 1 |  | n=425 0.83 (0.73, 0.94) | 0.004 |  |
|  |  | 1 or 2 | n=227 0.98 (0.73, 1.34) | 0.90 | n=64 0.83 (0.70, 0.99) | 0.04 | 0.54 |  | n=162 1.03 (0.72, 1.46) | 0.89 | n=72 0.91 (0.77, 1.08) | 0.29 | 0.82 |
|  | rs1799853 | 0 | n=1423 1 |  | n=371 0.86 (0.76, 0.97) | 0.02 |  |  | n=114 1 |  | n=90 0.93 (0.67, 1.28) | 0.65 |  |
|  |  | 1 or 2 | n=423 0.83 (0.66, 1.05) | 0.12 | n=119 0.79 (0.69, 0.89) | <0.001 | 0.36 |  | n=32 1.40 (0.57, 3.48) | 0.35 | n=19 0.82 (0.56, 1.21) | 0.32 | 0.25 |
| ***CCAT2*** | rs6983267 | 0 | n=568 1 |  | n=164 0.91 (0.74, 1.09) | 0.32 |  |  | n=427 1 |  | n=169 0.86 (0.71, 1.05) | 0.14 |  |
|  |  | 1 or 2 | n=1337 0.96 (0.77, 1.18) | 0.68 | n=334 0.86 (0.77, 0.94) | 0.002 | 0.41 |  | n=960 0.79 (0.62, 1.02) | 0.07 | n=402 0.86 (0.77, 0.95) | 0.004 | 0.56 |
| ***Intergenic* 20p12** | rs961253 | 0 | n=752 1 |  | n=195 0.78 (0.66, 0.92) | 0.003 |  |  | n=568 1 |  | n=223 0.80 (0.67, 0.95) | 0.01 |  |
|  |  | 1 or 2 | n=1160 1.07 (0.89, 1.32) | 0.51 | n=304 0.95 (0.86, 1.04) | 0.25 | 0.17 |  | n=822 0.96 (0.77, 1.22) | 0.76 | n=348 0.90 (0.82, 1.00) | 0.05 | 0.28 |
| ***ODC1*** | rs28362380 | 0 | n=1501 1 |  | n=399 0.81 (0.72, 0.91) | <0.001 |  |  | n=1126 1 |  | n=471 0.84 (0.74, 0.95) | 0.004 |  |
|  |  | 1 or 2 | n=327 0.95 (0.74, 1.23) | 0.72 | n=77 0.92 (0.78, 1.08) | 0.33 | 0.36 |  | n=266 1.11 (0.83, 1.49) | 0.48 | n=100 0.98 (0.85, 1.14) | 0.80 | 0.62 |
|  | rs11694911 | 0 | n=1477 1 |  | n=384 0.83 (0.74, 0.94) | 0.003 |  |  | n=1095 1 |  | n=457 0.85 (0.75, 0.96) | 0.007 |  |
|  |  | 1 or 2 | n=372 0.79 (0.63, 1.01) | 0.06 | n=99 0.78 (0.68, 0.90) | 0.001 | 0.58 |  | n=297 0.82 (0.62, 1.08) | 0.15 | n=114 0.92 (0.80, 1.05) | 0.22 | 0.24 |
|  | rs2430420 | 0 | n=821 1 |  | n=206 0.85 (0.72, 0.99) | 0.04 |  |  | - | - | - | - | - |
|  |  | 1 or 2 | n=1028 0.98 (0.80, 1.18) | 0.83 | n=280 0.88 (0.80, 0.97) | 0.01 | 0.95 |  | - | - | - | - | - |
|  | rs2302615 | 0 | n=969 1 |  | n=260 0.83 (0.72, 0.97) | 0.01 |  |  | - | - | - | - | - |
|  |  | 1 or 2 | n=799 0.86 (0.71, 1.06) | 0.13 | n=213 0.83 (0.75, 0.93) | 0.001 | 0.94 |  | - | - | - | - | - |
| ***PAFAH1B2*** | rs4936367 | 0 | n=1481 1 |  | n=393 0.79 (0.70, 0.89) | <0.001 |  |  | n=1114 1 |  | n=456 0.84 (0.75, 0.95) | 0.006 |  |
|  |  | 1 or 2 | n=372 0.99 (0.77, 1.26) | 0.93 | n=93 1.01 (0.87, 1.18) | 0.86 | **0.04** |  | n=278 0.82 (0.62, 1.09) | 0.17 | n=115 0.91 (0.79, 1.04) | 0.17 | 0.54 |
|  | rs7112513 | 0 | n=1521 1 |  | n=404 0.80 (0.71, 0.90) | <0.001 |  |  | n=1109 1 |  | n=453 0.84 (0.74, 0.95) | 0.004 |  |
|  |  | 1 or 2 | n=391 1.01 (0.79, 1.28) | 0.95 | n=95 1.00 (0.86, 1.17) | 0.96 | 0.08 |  | n=279 0.79 (0.60, 1.05) | 0.10 | n=115 0.91 (0.79, 1.04) | 0.16 | 0.44 |
| ***PTGS1*** | rs3842787 | 0 | n=1152 1 |  | n=294 0.83 (0.72, 0.95) | 0.006 |  |  | n=1220 1 |  | n=478 0.88 (0.78, 0.99) | 0.04 |  |
|  |  | 1 or 2 | n=262 1.08 (0.81, 1.45) | 0.62 | n=75 0.99 (0.84, 1.17) | 0.90 | 0.39 |  | n=165 1.02 (0.72, 1.55) | 0.92 | n=92 0.84 (0.72, 0.98) | 0.02 | 0.24 |
| ***PTGS2*** | rs4648310 | 0 | n=1726 1 |  | n=458 0.82 (0.74, 0.92) | 0.001 |  |  | n=1176 1 |  | n=470 0.87 (0.78, 0.98) | 0.02 |  |
|  |  | 1 or 2 | n=120 0.97 (0.66, 1.45) | 0.89 | n=26 0.87 (0.67, 1.14) | 0.33 | 0.97 |  | n=75 1.09 (0.66, 1.80) | 0.73 | n=31 0.72 (0.55, 0.94) | 0.02 | 0.12 |
|  | rs20417 | 0 | n=1342 1 |  | n=342 0.80 (0.71, 0.91) | 0.001 |  |  | n=951 1 |  | n=399 0.90 (0.79, 1.03) | 0.12 |  |
|  |  | 1 or 2 | n=508 1.08 (0.87, 1.36) | 0.51 | n=146 0.94 (0.83, 1.06) | 0.32 | 0.43 |  | n=439 0.99 (0.78, 1.27) | 0.95 | n=172 0.85 (0.75, 0.95) | 0.005 | 0.35 |
|  | rs2745557 | 0 | n=1247 1 |  | n=345 0.84 (0.74, 0.96) | 0.01 |  |  | n=835 1 |  | n=320 0.87 (0.75, 1.01) | 0.07 |  |
|  |  | 1 or 2 | n=556 0.90 (0.72, 1.12) | 0.33 | n=131 0.83 (0.73, 0.94) | 0.004 | 0.69 |  | n=404 1.32 (1.01, 1.71) | 0.04 | n=140 0.95 (0.83, 1.08) | 0.41 | 0.83 |
|  | rs5277 | 0 | n=1334 1 |  | n=347 0.85 (0.75, 0.97) | 0.01 |  |  | - | - | - | - | - |
|  |  | 1 or 2 | n=522 1.22 (0.98, 1.53) | 0.07 | n=140 0.87 (0.77, 0.99) | 0.03 | 0.20 |  | - | - | - | - | - |
|  | rs5275 | 0 | - | - | - | - | - |  | n=580 1 |  | n=232 0.87 (0.74, 1.04) | 0.12 |  |
|  |  | 1 or 2 | - | - | - | - | - |  | n=748 0.83 (0.65, 1.04) | 0.11 | n=313 0.85 (0.77, 0.95) | 0.003 | 0.36 |
| ***UGT1A6*** | rs1105879 | 0 | n=867 1 |  | n=237 0.78 (0.67, 0.91) | 0.001 |  |  | n=598 1 |  | n=234 0.81 (0.68, 0.96) | 0.01 |  |
|  |  | 1 or 2 | n=1041 1.02 (0.85, 1.25) | 0.86 | n=263 0.95 (0.86, 1.05) | 0.34 | 0.10 |  | n=791 0.93 (0.74, 1.17) | 0.56 | n=337 0.91 (0.82, 1.01) | 0.07 | 0.18 |
|  | rs2070959 | 0 | n=931 1 |  | n=253 0.77 (0.66, 0.89) | <0.001 |  |  | n=638 1 |  | n=246 0.80 (0.68, 0.95) | 0.009 |  |
|  |  | 1 or 2 | n=975 1.04 (0.86, 1.27) | 0.71 | n=247 0.97 (0.87, 1.07) | 0.51 | **0.05** |  | n=752 0.87 (0.69, 1.09) | 0.24 | n=325 0.90 (0.81, 0.99) | 0.04 | 0.17 |
| ***IL16*** | rs16973225 | 0 | n=1584 1 |  | n=398 0.85 (0.75, 0.96) | 0.008 |  |  | - | - | - | - | - |
|  |  | 1 or 2 | n=185 0.89 (0.65, 1.25) | 0.49 | n=60 0.83 (0.69, 0.99) | 0.04 | 0.70 |  | - | - | - | - | - |
|  | rs12910333 | 0 | n=927 1 |  | n=248 0.81 (0.69, 0.94) | 0.005 |  |  | n=697 1 |  | n=291 0.91 (0.78, 1.06) | 0.23 |  |
|  |  | 1 or 2 | n=847 0.88 (0.72, 1.08) | 0.22 | n=208 0.90 (0.80, 1.00) | 0.06 | 0.30 |  | n=694  0.96 (0.76, 1.20) | 0.70 | n=280 0.88 (0.79, 0.98) | 0.02 | 0.67 |
| ***IKBKB*** | rs11986055 | 0 | n=1661 1 |  | n=447 0.88 (0.78, 0.98) | 0.02 |  |  | n=1280 1 |  | n=534 0.86 (0.77, 0.97) | 0.01 |  |
|  |  | 1 or 2 | n=133 1.42 (0.95, 2.14) | 0.09 | n=28 0.86 (0.66, 1.11) | 0.24 | 0.21 |  | n=110 0.97 (0.63, 1.47) | 0.87 | n=37 0.93 (0.74, 1.17) | 0.52 | 0.74 |
|  | rs10958713 | 0 | n=755 1 |  | n=208 0.86 (0.73, 1.02) | 0.07 |  |  | n=591 1 |  | n=238 0.87 (0.74, 1.04) | 0.12 |  |
|  |  | 1 or 2 | n=1047 1.00 (0.81, 1.23) | 0.97 | n=262 0.90 (0.81, 0.99) | 0.04 | 0.79 |  | n=801 0.94 (0.75, 1.19) | 0.61 | n=332 0.88 (0.80, 0.98) | 0.02 | 0.58 |
|  | rs5029748 | 0 | - | - | - | - | - |  | n=704 1 |  | n=267 0.80 (0.68, 0.94) | 0.007 |  |
|  |  | 1 or 2 | - | - | - | - | - |  | n=547 0.92 (0.73, 1.17) | 0.50 | n=233 0.90 (0.81, 1.01) | 0.08 | 0.69 |
|  | rs6474387 | 0 | - | - | - | - | - |  | n=137 1 |  | n=93 0.98 (0.72, 1.32) | 0.88 |  |
|  |  | 1 or 2 | - | - | - | - | - |  | n=15 1.36 (0.42, 4.43) | 0.61 | n=18 0.73 (0.49, 1.08) | 0.12 | 0.12 |
| ***NCF4*** | rs5995355 | 0 | n=1564 1 |  | n=406 0.82 (0.73, 0.92) | 0.001 |  |  | n=1208 1 |  | n=512 0.88 (0.78, 0.98) | 0.03 |  |
|  |  | 1 or 2 | n=217 1.01 (0.75, 1.39) | 0.97 | n=61 1.07 (0.88, 1.30) | 0.49 | 0.08 |  | n=183 1.66 (1.16, 2.37) | 0.006 | n=59 0.91 (0.76, 1.09) | 0.30 | 0.07 |
| ***ALOX15*** | rs2619112 | 0 | n=506 1 |  | n=129 0.73 (0.60, 0.90) | 0.004 |  |  | n=390 1 |  | n=159 0.94 (0.76, 1.16) | 0.57 |  |
|  |  | 1 or 2 | n=1253 0.98 (0.78, 1.22) | 0.88 | n=330 0.90 (0.81, 1.00) | 0.05 | 0.20 |  | n=1002 1.16 (0.90, 1.49) | 0.24 | n=412 0.92 (0.83, 1.03) | 0.14 | 0.47 |
| ***NFKB*** | rs230490 | 0 | n=548 1 |  | n=143 0.96 (0.79, 1.18) | 0.70 |  |  | n=485 1 |  | n=178 0.76 (0.63, 0.92) | 0.006 |  |
|  |  | 1 or 2 | n=1225 1.10 (0.89, 1.38) | 0.40 | n=324 0.90 (0.82, 1.00) | 0.05 | 0.16 |  | n=907 0.94 (0.74, 1.19) | 0.61 | n=393 0.92 (0.83, 1.02) | 0.11 | 0.47 |
| ***MGST1*** | rs2965667 | 0 | n=1450 1 |  | n=368 0.86 (0.75, 0.97) | 0.02 |  |  | - | - | - | - | - |
|  |  | 1 or 2 | n=116 1.15 (0.75, 1.82) | 0.54 | n=27 0.78 (0.60, 1.03) | 0.06 | 0.24 |  | - | - | - | - | - |
| ***IL23R*** | rs6683455 | 0 | - | - | - | - | - |  | n=940 1 |  | n=382 0.82 (0.72, 0.93) | 0.003 |  |
|  |  | 1 or 2 | - | - | - | - | - |  | n=301 0.96 (0.73, 1.27) | 0.77 | n=117 0.87 (0.76, 1.00) | 0.05 | 0.66 |
| ***PGDH*** | rs7349744 | 0 | - | - | - | - | - |  | n=154 1 |  | n=86 0.79 (0.56, 1.11) | 0.17 |  |
|  |  | 1 or 2 | - | - | - | - | - |  | n=140 1.04 (0.55, 1.95) | 0.91 | n=95 1.05 (0.84, 1.32) | 0.66 | 0.54 |
| ***FLAP*** | rs17239025 | 0 | - | - | - | - | - |  | n=144 1 |  | n=97 0.91 (0.68, 1.23) | 0.54 |  |
|  |  | 1 or 2 | - | - | - | - | - |  | n=9 0.53 (0.12, 2.41) | 0.41 | n=14 0.68 (0.42, 1.11) | 0.12 | 0.93 |

+*P*-value for association adjusted for age, sex and study site.

**P*-value for interaction between SNP variant allele, aspirin use and colorectal cancer risk calculated using Likelihood ratio test. *P-*value is adjusted for age, sex and study site.

OR, Odds Ratio

CI, Confidence Interval

n, Number of subjects
