# Supplementary material for: Interaction between polymorphisms in aspirin metabolic pathways, regular aspirin use and colorectal cancer risk: A case-control study in unselected white European populations
Source: PLoS One. 2018 Feb 9;13(2):e0192223. doi: 10.1371/journal.pone.0192223 (PMC5806861; doi:10.1371/journal.pone.0192223)
Supplement: S12 Table — +P-value for association adjusted for age, sex and study site. *P-value for interaction between SNP variant allele, aspirin use and colorectal cancer risk calculated using Likelihood ratio test. P-value is adjusted for age, sex and study site. ~P-value for Cochran’s Q-test for heterogeneity OR, Odds Ratio CI, Confidence Interval. (DOCX) [file pone.0192223.s015.docx]

S12 Table: Meta-analysis of interaction between SNP variant allele, aspirin only use and colorectal cancer.

| **Gene name** | **SNP ID** | **Non-users** | | **Aspirin users** | | ***P*-value for interaction*** | **I-squared (%)** | ***P-*value^~^** |
| --- | --- | --- | --- | --- | --- | --- | --- | --- |
|  |  | **OR (95% CI)** | ***P*-value+** | **OR (95% CI)** | ***P*-value+** |  |  |  |
| ***MDR1*** | rs1045642 | Reference |  | 0.82 (0.71, 00.95) | 0.008 |  |  |  |
|  |  | 1.01 (0.86, 1.20) | 0.87 | 0.92 (0.85, 0.99) | 0.02 | 0.59 | 0 | 0.57 |
| ***CYP2C9*** | rs1057910 | Reference |  | 0.83 (0.76, 0.90) | <0.001 |  |  |  |
|  |  | 1.02 (0.80, 1.29) | 0.88 | 0.85 (0.74, 0.98) | 0.02 | 0.59 | 0 | 0.39 |
|  | rs1799853 | Reference |  | 0.84 (0.75, 0.95) | 0.006 |  |  |  |
|  |  | 0.89 (0.64, 1.25) | 0.52 | 0.76 (0.67, 0.87) | <0.001 | 0.15 | 0 | 0.46 |
| ***CCAT2*** | rs6983267 | Reference |  | 0.87 (0.75, 1.00) | 0.05 |  |  |  |
|  |  | 0.86 (0.73, 1.02) | 0.08 | 0.84 (0.78, 0.91) | <0.001 | 0.80 | 19.2 | 0.27 |
| ***Intergenic* 20p12** | rs961253 | Reference |  | 0.79 (0.70, 0.90) | <0.001 |  |  |  |
|  |  | 1.06 (0.90, 1.24) | 0.51 | 0.92 (0.85, 0.98) | 0.02 | 0.24 | 0 | 0.74 |
| ***ODC1*** | rs28362380 | Reference |  | 0.81 (0.75, 0.89) | <0.001 |  |  |  |
|  |  | 1.02 (0.84, 1.24) | 0.84 | 0.95 (0.85, 1.06) | 0.39 | 0.29 | 0 | 0.69 |
|  | rs11694911 | Reference |  | 0.83 (0.76, 0.90) | <0.001 |  |  |  |
|  |  | 0.80 (0.67, 0.96) | 0.02 | 0.84 (0.71, 1.00) | 0.05 | 0.72 | 32.3 | 0.22 |
|  | rs2430420 | Reference |  | 0.82 (0.69, 0.97) | 0.02 |  |  |  |
|  |  | 0.98 (0.80, 1.20) | 0.84 | 0.87 (0.78, 0.96) | 0.005 | 0.69 | - | - |
|  | rs2302615 | Reference |  | 0.81 (0.69, 0.94) | 0.005 |  |  |  |
|  |  | 0.84 (0.69, 1.04) | 0.12 | 0.82 (0.74, 0.91) | <0.001 | 0.57 | - | - |
| ***PAFAH1B2*** | rs4936367 | Reference |  | 0.80 (0.72, 0.89) | <0.001 |  |  |  |
|  |  | 0.90 (0.74, 1.09) | 0.26 | 0.95 (0.85, 1.05) | 0.31 | 0.13 | 41.8 | 0.19 |
|  | rs7112513 | Reference |  | 0.80 (0.74, 0.88) | <0.001 |  |  |  |
|  |  | 0.89 (0.72, 1.09) | 0.25 | 0.94 (0.85, 1.05) | 0.26 | 0.07 | 0 | 0.37 |
| ***PTGS1*** | rs3842787 | Reference |  | 0.84 (0.76, 0.93) | 0.001 |  |  |  |
|  |  | 0.99 (0.79, 1.26) | 0.95 | 0.87 (0.77, 0.97) | 0.01 | 0.47 | 0 | 0.34 |
| ***PTGS2*** | rs4648310 | Reference |  | 0.83 (0.77, 0.90) | <0.001 |  |  |  |
|  |  | 0.99 (0.72, 1.35) | 0.93 | 0.77 (0.64, 0.92) | 0.005 | 0.20 | 0 | 0.35 |
|  | rs20417 | Reference |  | 0.84 (0.74, 0.96) | 0.01 |  |  |  |
|  |  | 1.06 (0.90, 1.25) | 0.50 | 0.89 (0.81, 0.97) | 0.01 | 0.79 | 0 | 0.30 |
|  | rs2745557 | Reference |  | 0.84 (0.76, 0.93) | 0.001 |  |  |  |
|  |  | 1.09 (0.75, 1.57) | 0.66 | 0.88 (0.76, 1.02) | 0.09 | 0.53 | 0 | 0.75 |
|  | rs5275 | Reference |  | 0.87 (0.74, 1.04) | 0.12 |  |  |  |
|  |  | 0.83 (0.65, 1.05) | 0.11 | 0.85 (0.77, 0.95) | 0.003 | 0.36 | - | - |
|  | rs5277 | Reference |  | 0.84 (0.74, 0.95) | 0.007 |  |  |  |
|  |  | 1.25 (0.99, 1.57) | 0.06 | 0.86 (0.76, 0.97) | 0.02 | 0.25 | - | - |
| ***UGT1A6*** | rs1105879 | Reference |  | 0.77 (0.69, 0.86) | <0.001 |  |  |  |
|  |  | 0.97 (0.83, 1.13) | 0.70 | 0.92 (0.86, 0.99) | 0.03 | **0.02** | 0 | 0.54 |
|  | rs2070959 | Reference |  | 0.77 (0.68, 0.86) | <0.001 |  |  |  |
|  |  | 0.95 (0.82, 1.11) | 0.54 | 0.92 (0.86, 0.99) | 0.03 | **0.01** | 0 | 0.44 |
| ***IL16*** | rs12910333 | Reference |  | 0.85 (0.74, 0.97) | 0.02 |  |  |  |
|  |  | 0.93 (0.80, 1.09) | 0.37 | 0.88 (0.82, 0.95) | 0.001 | 0.83 | 0 | 0.37 |
| ***IKBKB*** | rs11986055 | Reference |  | 0.86 (0.79, 0.93) | <0.001 |  |  |  |
|  |  | 1.16 (0.81, 1.65) | 0.42 | 0.89 (0.74, 1.05) | 0.17 | 0.57 | 23.6 | 0.25 |
|  | rs10958713 | Reference |  | 0.85 (0.75, 0.96) | 0.008 |  |  |  |
|  |  | 0.97 (0.83, 1.13) | 0.68 | 0.88 (0.82, 0.95) | 0.001 | 0.85 | 0 | 0.54 |
|  | rs5029748 | Reference |  | 0.80 (0.68, 0.94) | 0.007 |  |  |  |
|  |  | 0.92 (0.73, 1.17) | 0.50 | 0.91 (0.81, 1.01) | 0.08 | 0.69 | - | - |
|  | rs6474387 | Reference |  | 0.98 (0.72, 1.32) | 0.88 |  |  |  |
|  |  | 1.36 (0.42, 4.43) | 0.61 | 0.73 (0.49, 1.08) | 0.12 | 0.13 | - | - |
| ***NCF4*** | rs5995355 | Reference |  | 0.84 (0.76, 0.92) | <0.001 |  |  |  |
|  |  | 1.30 (0.81, 2.07) | 0.28 | 0.97 (0.84, 1.13) | 0.72 | 0.95 | 82.6 | 0.02 |
| ***ALOX15*** | rs2619112 | Reference |  | 0.82 (0.62, 1.08) | 0.16 |  |  |  |
|  |  | 1.05 (0.88, 1.26) | 0.57 | 0.90 (0.84, 0.98) | 0.009 | 0.99 | 7.6 | 0.30 |
| ***NFKB*** | rs230490 | Reference |  | 0.84 (0.69, 1.03) | 0.10 |  |  |  |
|  |  | 1.03 (0.86, 1.25) | 0.73 | 0.91 (0.84, 0.98) | 0.009 | 0.68 | 67.6 | 0.08 |
| ***MGST1*** | rs2965667 | Reference |  | 0.84 (0.73, 0.96) | 0.01 |  |  |  |
|  |  | 1.15 (0.72, 1.82) | 0.56 | 0.79 (0.60, 1.03) | 0.08 | 0.31 | - | - |
| ***IL23R*** | rs6683455 | Reference |  | 0.82 (0.72, 0.93) | 0.003 |  |  |  |
|  |  | 0.96 (0.72, 1.26) | 0.75 | 0.87 (0.76, 1.00) | 0.05 | 0.66 | - | - |
| ***PGDH*** | rs7349744 | Reference |  | 0.79 (0.56, 1.11) | 0.17 |  |  |  |
|  |  | 1.04 (0.55, 1.95) | 0.91 | 1.05 (0.84, 1.32) | 0.66 | 0.54 | - | - |
| ***FLAP*** | rs17239025 | Reference |  | 0.91 (0.68, 1.23) | 0.54 |  |  |  |
|  |  | 0.53 (0.12, 2.41) | 0.41 | 0.68 (0.42, 1.11) | 0.12 | 0.93 | - | - |

+*P*-value for association adjusted for age, sex and study site.

**P*-value for interaction between SNP variant allele, aspirin use and colorectal cancer risk calculated using Likelihood ratio test. *P-*value is adjusted for age, sex and study site.

~*P-*value for Cochran’s Q-test for heterogeneity

OR, Odds Ratio

CI, Confidence Interval
