# Supplementary material for: Interaction between polymorphisms in aspirin metabolic pathways, regular aspirin use and colorectal cancer risk: A case-control study in unselected white European populations
Source: PLoS One. 2018 Feb 9;13(2):e0192223. doi: 10.1371/journal.pone.0192223 (PMC5806861; doi:10.1371/journal.pone.0192223)
Supplement: S1 Fig — (DOCX) [file pone.0192223.s017.docx]

**
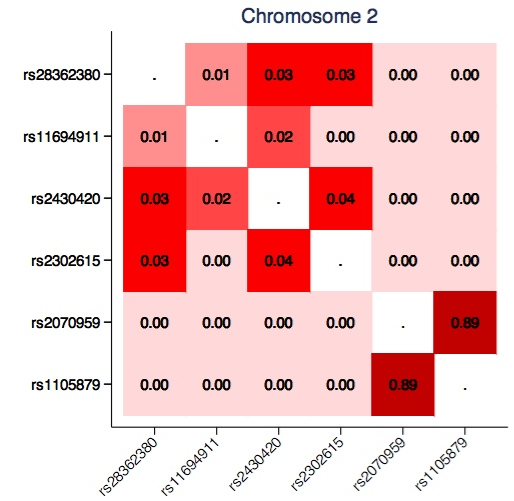
**
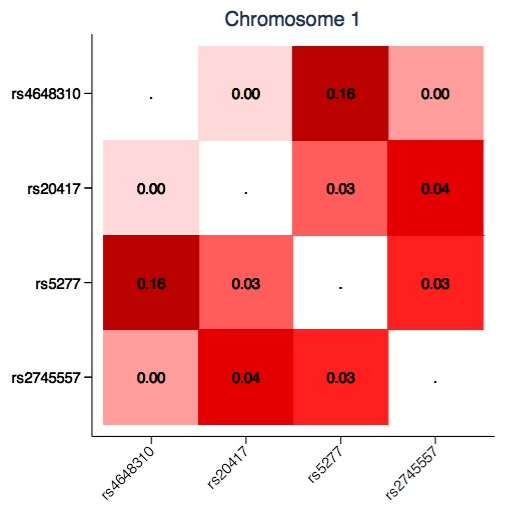
**A B**

**C D**


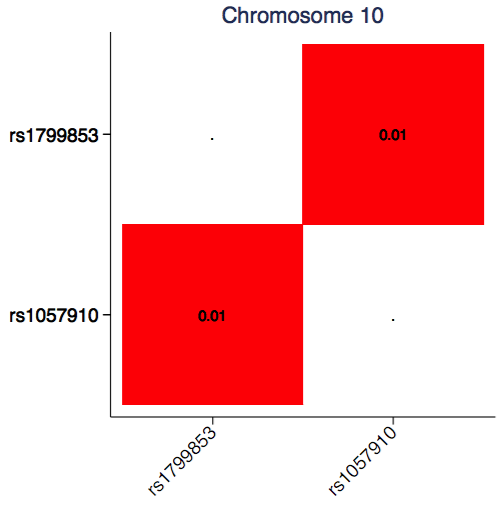

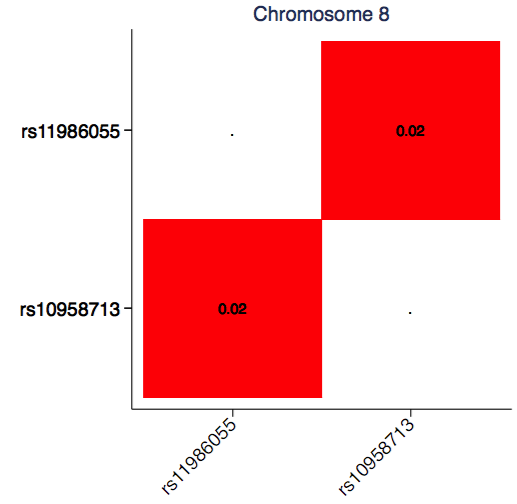


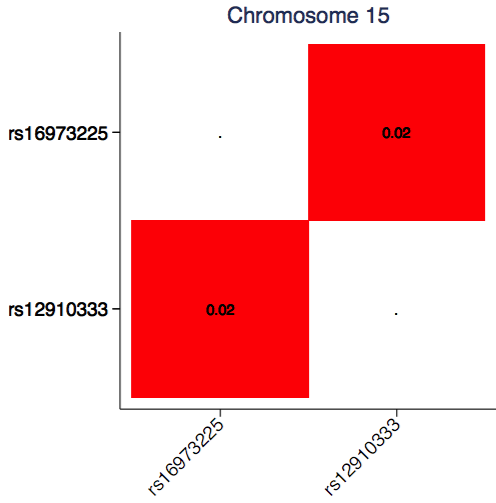
**
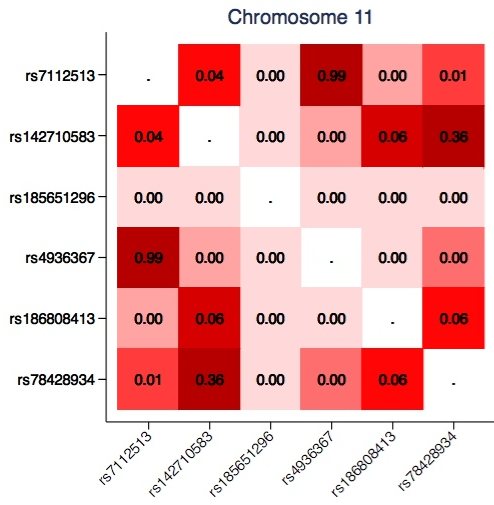
E F**

S1 Fig: Linkage disequilibrium (R^2^) heat maps for SNPs in the UK-Colorectal Cancer Study Group dataset.
