# Supplementary material for: Interaction between polymorphisms in aspirin metabolic pathways, regular aspirin use and colorectal cancer risk: A case-control study in unselected white European populations
Source: PLoS One. 2018 Feb 9;13(2):e0192223. doi: 10.1371/journal.pone.0192223 (PMC5806861; doi:10.1371/journal.pone.0192223)
Supplement: S2 Fig — (DOCX) [file pone.0192223.s018.docx]

**
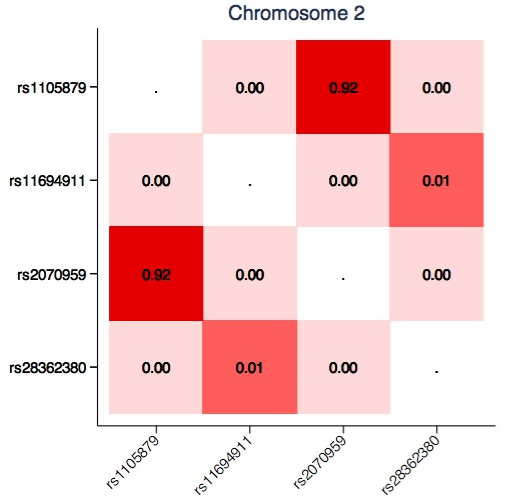
A B**

**
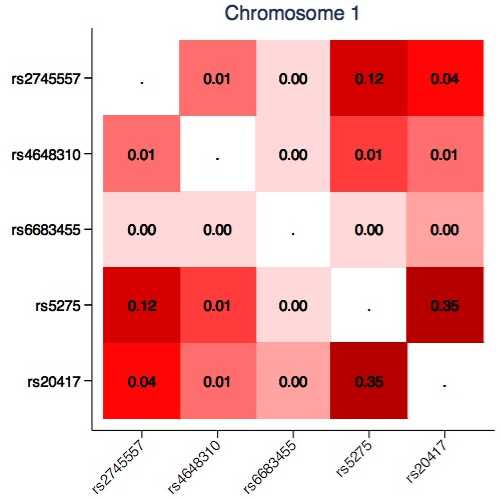
**

**C D**

**
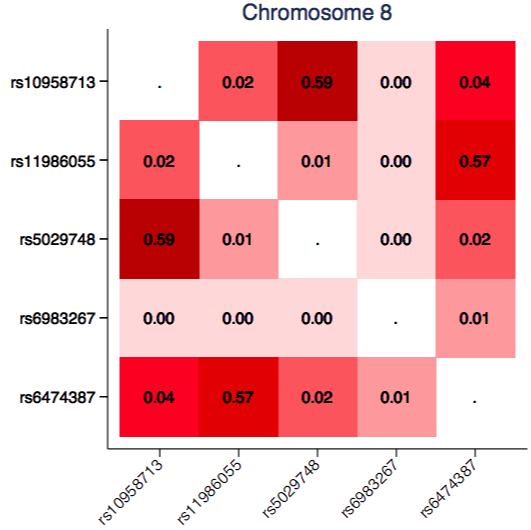
**

**
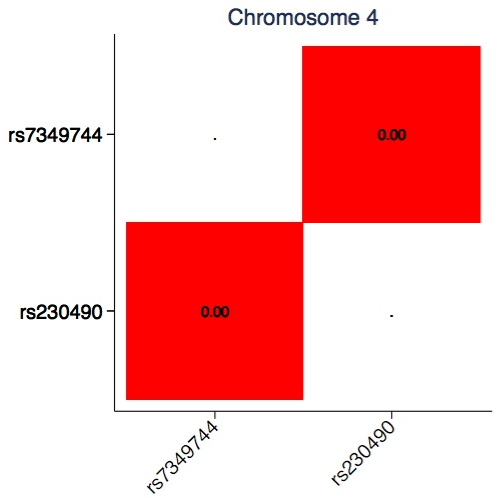
**

**E F**


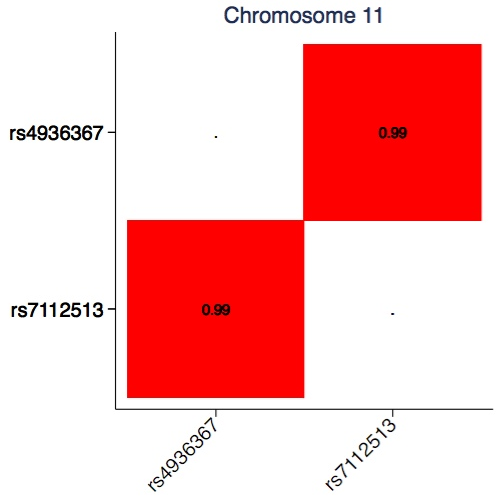

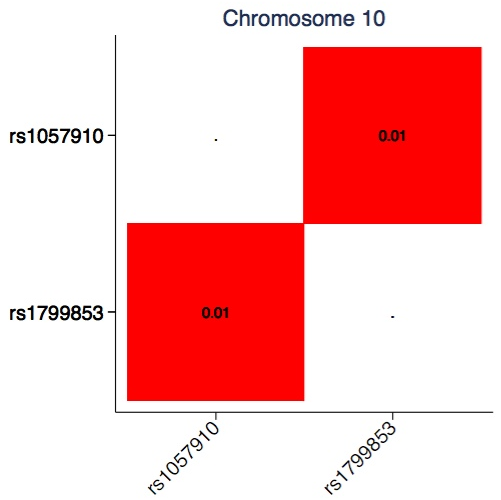


**S2 Fig: Linkage disequilibrium (R^2^) heat maps for SNPs in the NIH-Colon Cancer Family Registry dataset.**
